# *De novo* Transcriptome Sequencing and Development of Abscission Zone-Specific Microarray as a New Molecular Tool for Analysis of Tomato Organ Abscission

**DOI:** 10.3389/fpls.2015.01258

**Published:** 2016-01-14

**Authors:** Srivignesh Sundaresan, Sonia Philosoph-Hadas, Joseph Riov, Raja Mugasimangalam, Nagesh A. Kuravadi, Bettina Kochanek, Shoshana Salim, Mark L. Tucker, Shimon Meir

**Affiliations:** ^1^Department of Postharvest Science of Fresh Produce, Agricultural Research Organization, The Volcani CenterBet-Dagan, Israel; ^2^The Robert H. Smith Faculty of Agriculture, Food and Environment, The Robert H. Smith Institute of Plant Sciences and Genetics in Agriculture, The Hebrew University of JerusalemRehovot, Israel; ^3^Department of Bioinformatics, QTLomics Technologies Pvt. LtdBangalore, India; ^4^Soybean Genomics and Improvement Laboratory, United States Department of Agriculture, Agricultural Research ServiceBeltsville, MD, USA

**Keywords:** auxin, ethylene, flower pedicel abscission, leaf petiole abscission, oligonucleotide microarray, RNA-Sequencing, tomato (*Solanum lycopersicum*), transcriptome

## Abstract

Abscission of flower pedicels and leaf petioles of tomato (*Solanum lycopersicum*) can be induced by flower removal or leaf deblading, respectively, which leads to auxin depletion, resulting in increased sensitivity of the abscission zone (AZ) to ethylene. However, the molecular mechanisms that drive the acquisition of abscission competence and its modulation by auxin gradients are not yet known. We used RNA-Sequencing (RNA-Seq) to obtain a comprehensive transcriptome of tomato flower AZ (FAZ) and leaf AZ (LAZ) during abscission. RNA-Seq was performed on a pool of total RNA extracted from tomato FAZ and LAZ, at different abscission stages, followed by *de novo* assembly. The assembled clusters contained transcripts that are already known in the Solanaceae (SOL) genomics and NCBI databases, and over 8823 identified novel tomato transcripts of varying sizes. An AZ-specific microarray, encompassing the novel transcripts identified in this study and all known transcripts from the SOL genomics and NCBI databases, was constructed to study the abscission process. Multiple probes for longer genes and key AZ-specific genes, including antisense probes for all transcripts, make this array a unique tool for studying abscission with a comprehensive set of transcripts, and for mining for naturally occurring antisense transcripts. We focused on comparing the global transcriptomes generated from the FAZ and the LAZ to establish the divergences and similarities in their transcriptional networks, and particularly to characterize the processes and transcriptional regulators enriched in gene clusters that are differentially regulated in these two AZs. This study is the first attempt to analyze the global gene expression in different AZs in tomato by combining the RNA-Seq technique with oligonucleotide microarrays. Our AZ-specific microarray chip provides a cost-effective approach for expression profiling and robust analysis of multiple samples in a rapid succession.

## Introduction

Abscission is a systematically regulated process in plant development, by which subtended organs, leaves, flowers, fruit, and seed, separate from the parent plant in response to various physiological cues. This process is required to recycle nutrients for continuous growth, develop appropriate organs, survive diseases, and facilitate reproduction (Addicott, 1982; Sexton and Roberts, 1982; Roberts et al., 2002; Lewis et al., 2006). Since the domestication of crops was started, a great emphasis has been put forth on selection for abrupted abscission to improve crop qulaity and yield. For example, reduced seed shaterring in rice (Li et al., 2006), wheat (Tanno and Willcox, 2006), maize (Doebley, 2004), and fruit tree species (Bangerth, 2000) was obtained. In an agricultural perspective, both enhanced and delayed abscission are highly relevant for growers.

In plants, the abscission process occurs at a predetermined region called abscission zone (AZ), composed of few layers of small and dense cytoplasmic cells, which lack large vacuoles and any maturation characteristics (Osborne and Morgan, 1989), resembling undifferentiated cells (Van Nocker, 2009). AZ cells belong to type II cells, in which extended growth can be enhanced by ethylene, but not by auxin (McManus, 2008), conferring their meristematic potential (Roberts et al., 2000). Physiological studies revealed that ethylene and auxin control the cell separation process. The abscission process is initiated or timed by changes in the auxin gradient across the AZ, and is acceltrated by ethylene (Roberts et al., 2002; McManus, 2008; Meir et al., 2010). The four key steps in the abscission process are: differentiation of the AZ, acquisition of the competence to respond to abscission signals, execution of organ abscission, and formation of a protective layer (Meir et al., 2011; Wang et al., 2013). During the late abscission stages, the cell wall and middle lamella are the major targets for degradation, which is operated by many cell wall modifiying enzymes, including polygalacturonases (PGs), xyloglucan endotransglucosylase/ hydrolase (XTH), β-1,4-glucanase (cellulase, Cel), and expansins (EXP) (Lashbrook et al., 1994; del Campillo and Bennett, 1996; Cho and Cosgrove, 2000; Taylor and Whitelaw, 2001; Roberts et al., 2002; Ogawa et al., 2009; Meir et al., 2010).

Tomato (*Solanum lycopersicum*) is one of the most important vegetable crops, whose genome sequence was recently published (The Tomato Genome Consortium, 2012). It serves as a model crop for studying fruit development (Klee and Giovannoni, 2011) and abscission, as it posses a distinct joint-like structure in the AZ of the flower pedicels. The molecular mechanisms underlying the abscission progress in tomato are still evolving, even though the abscission physiology was studied long ago (Sexton and Roberts, 1982; Bleecker and Patterson, 1997; Roberts et al., 2000). The genes affecting AZ development have been identified by studying abscission-impaired mutants, such as *jointless, jointless2, macrocalyx, blind* (*bl*), *and lateral supressor* (*ls*) (Butler, 1936; Rick, 1956; Schumacher et al., 1999; Mao et al., 2000; Szymkowiak and Irish, 2006; Shalit et al., 2009; Nakano et al., 2012; Liu et al., 2014). However, information regarding the expression of AZ-associated genes in tomato is still lacking.

Micro/oligo nucleotide arrays have been used until recently to study semi-global gene expression in AZs of citrus leaves (Agustí et al., 2008, 2009, 2012) and shoot tips (Zhang et al., 2014), tomato flowers (Meir et al., 2010, 2011; Nakano et al., 2012, 2013; Wang et al., 2013; Ma et al., 2015), Arabidopsis stamen (Cai and Lashbrook, 2008), and apple fruit and fruitlets (Botton et al., 2011; Zhu et al., 2011). The information on gene specific resources of tomato AZs during the abscission process obtained by microarrays is limited. The new era of high throughput next generation sequencing (NGS) technologies and bioinformatics tools to analyze and integrate the vast data, led to a significant rapid progress in the genomic research. RNA-Sequencing (RNA-Seq) involves direct sequencing of complementary DNAs (cDNAs), followed by mapping of reads to the reference genome or gene sets, to obtain a direct information from transcribed regions (Wang et al., 2009), gene expression profiles, and polymorphism detection in the genome. RNA-Seq provides a more comprehensive understanding of the transcriptome at a specific developmental stage of a tissue (Marioni et al., 2008; Wang et al., 2009; Parchman et al., 2010; Mäder et al., 2011), and an ability to detect novel transcripts, sense and antisense transcripts, single nucleotide polymorphism, small RNAs, alternate splice transcripts, and transcription initiation sites (Ozsolak and Milos, 2011). In comparison to other technologies, such as microarrays and Sanger-based sequencing technologies, RNA-Seq has additional advantages in terms of speed, depth, and accuracy. Recently, few RNA-Seq studies for studying the abscission process were carried out in various plant systems, such as olive (Gil-Amado and Gomez-Jimenez, 2013; Parra et al., 2013) and melon (Corbacho et al., 2013) fruit, Arabidopsis stamen AZ (Niederhuth et al., 2013), tomato flower AZ (FAZ) (Liu et al., 2014), and rose petals (Singh et al., 2013). However, a complete transcriptome study of the tomato FAZ and leaf AZ (LAZ) at various stages of the abscission process was not carried out, because of the higher cost of RNA-Seq analysis as compared to microarray. Considering the clear advantages of the RNA-Seq technology, the aim of the present research was to study the tomato FAZ and LAZ transcriptome, using RNA-Seq, followed by *de novo* transcriptome assembly and annotation.

In the last few years, customized-made expression arrays became more affordable to the scientific community due to the number of total features, complete coverage of genomic regions, and automated data analysis of microarrays. The information regarding the new transcripts, such as unannotated transcripts, splice variants, naturally occurring antisense transcripts (NATs), and novel genes in particular tissues, is less emphasized on the traditional arrays (Bertone et al., 2005; Mockler et al., 2005). More complexity is added due to methylation sites on the 3′ end that might interfere with transcription initiation and termination (Carninci et al., 2005, 2006). To overcome these problems, new customized microarray approaches for various needs, such as tilling arrays were developed, (Johnson et al., 2005). The custom-made arrays have a complete control of the number, expression, and distribution of probes specific to the studied system. In order to achieve higher hybridization and quality data, many considerations have to be taken while designing the customized microarrays, including probe length, density, melting temperatures, placement, level of cross hybridization, complexity, and mismatch levels to achieve the consensus property (Mei et al., 2003). The RNA-Seq information will be a useful tool to update the design of microarray probes for transcriptome analysis of large samples (Bellin et al., 2009).

In the present study, we used the Illumina sequencing technology to obtain a comprehensive transcriptome profile of the tomato FAZ and LAZ pooled samples taken at of various time points during organ abscission induced by auxin depletion, thereby expanding the tomato transcript catalog. We focused on comparing the transcriptomes generated from the FAZ and the LAZ tissues to establish the divergences and similarities in their transcriptional networks, and particularly to characterize the biological processes and transcriptional regulators enriched in gene clusters that are differentially regulated in these two AZs. The RNA-Seq data were used as a major source to design an AZ-specific microarray for tomato abscission studies. Additionally, in this chip the probes were designed in both sense and antisense orientations for transcripts, which enable future analyses of expression profiles of NATs in the AZs. The unique design of this chip allowed us to accurately quantify global changes in the transcriptome of the tomato AZs during the abscission process. Results from this study will help to identify target genes for further understanding and manipulating of abscission, as well as markers for breeding. We are currently using this chip to quantify molecular shifts in gene expression in transgenic plants that display altered abscission phenotypes.

## Materials and methods

### Plant material and treatments

**Figure 1:**
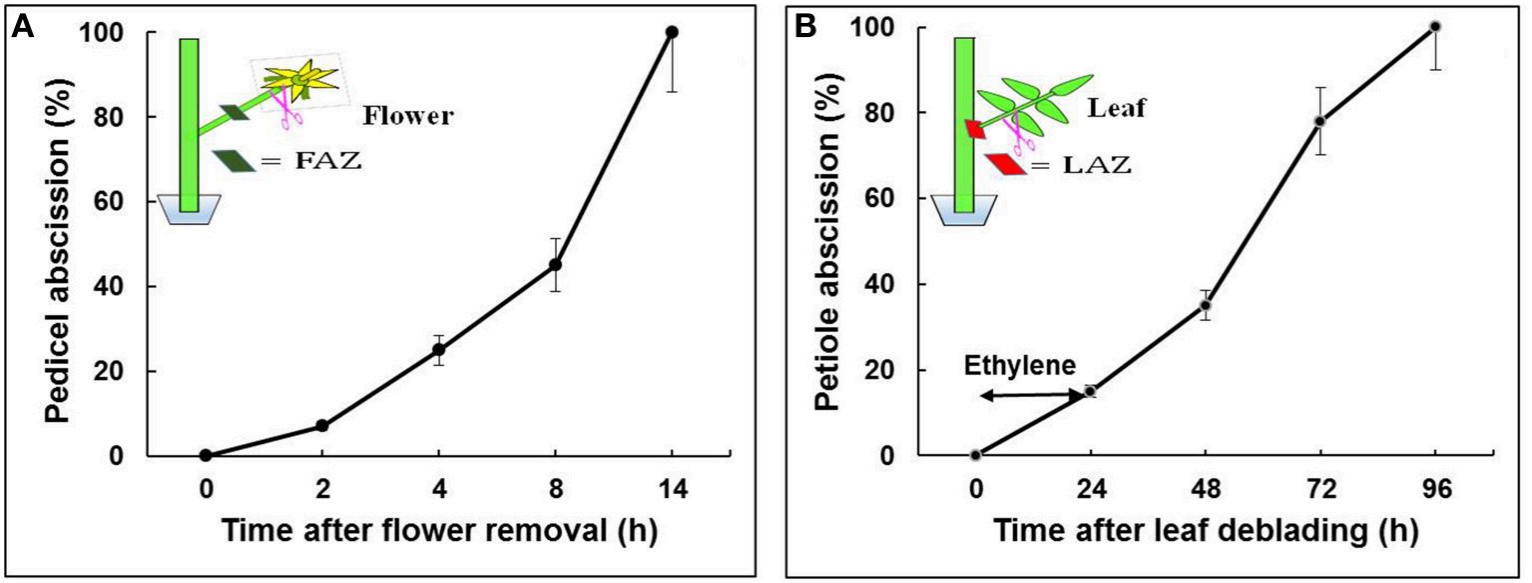
**Effect of flower removal (A) or leaf deblading and ethylene treatment (B) on the kinetics of pedicel and petiole abscission, respectively, in tomato explants**. Flowers and leaves were excised as indicated in the schematic illustrations. The debladed-leaf explants held in vials with water were prepared as previously described for the flower explants (Meir et al., 2010), and exposed to ethylene (10 μL L^−1^ for 24 h). The percentage of accumulated pedicel or petiole abscission were monitored at the indicated time intervals following organ removal. The results are means of four replicates (*n* = 30 explants) ± SE.

Tomato (*S. lycopersicum*) cv. “VF-36” flower bunches were harvested from greenhouse grown 4-month-old plants between 08:00 and 10:00 a.m. Bunches bearing at least 2–4 freshly open flowers were brought to the laboratory under high humidity conditions. All procedures were performed in a controlled observation room maintained at 20°C, 12 h photoperiod, and 60% RH. Closed young flower buds and senesced flowers were removed, and the stem ends of the bunches were trimmed. Groups of 2–3 flower explants were placed in vials containing double distilled water. The pedicel abscission assays were performed after flower removal, as previously described (Meir et al., 2010). For RNA extraction from the FAZ, 30 sections, <2-mm-thick, were excised, about 1 mm from each side of the visible AZ fracture. The samples were collected at predefined time points: 0 h—before flower removal, and 2, 4, 8, and 14 h after flower removal (Figure 1A).

Shoots containing at least 5–6 expanded leaves were brought to the laboratory under high humidity conditions. The shoot cut ends were trimmed, and the shoots were placed in jars containing double distilled water and incubated for 3 h to avoid dehydration before starting leaf deblading. The fully expanded leaves were debladed using a sharp razor by leaving a subtended 2-cm long petiole. To accelerate petiole abscission, the debladed leaf explants were exposed to 10 μL L^−1^ ethylene for 24 h in an air-tight chamber at 23°C. The number of abscising petioles was then recorded. The ethylene concentration was determined by a gas chromatograph (Varian 3300), equipped with an alumina column and a flame ionization detector, in a 5-mL air samples withdrawn from the chamber with a gas tight syringe. Tissue samples for RNA extraction were taken from 20 LAZ sections, as described above for the FAZ. The samples were collected at the following predefined time points: 0 h—before leaf deblading, and 24, 48, 72, and 96 h after leaf deblading and ethylene treatment (Figure 1B). All the collected FAZ and LAZ samples were placed in RNA-later solution for further analyses. Three biological replicates of equal tissue weight were taken from each sample for RNA extraction. Equal RNA quantities from all the time point samples of the FAZ or the LAZ were pooled to create one RNA sample of FAZ and one of the LAZ.

### RNA isolation and quality controls

Total RNA was extracted from the FAZ and the LAZ samples, using an Agilent plant RNA isolation mini kit (Agilent, USA). The concentration and purity of the RNA samples were examined by a Nanodrop instrument, and validated for quality by running an aliquot on a Bioanalyzer with RNA 6000 Nano Kit (Agilent Technologies, California, USA). Only samples having an RNA integrity number >8.0 were selected for cDNA preparation. RNA samples extracted from the FAZ and the LAZ at five specific time points were pooled to make individual transcriptome libraries for RNA-Seq study.

### Illumina sequencing and quality controls

Transcriptome libraries for sequencing were constructed according to the Illumina TruSeq RNA library protocol outlined in “TruSeq RNA Sample Preparation Guide” (Part # 15008136; Rev. A, Illumina, USA). Briefly, mRNA was purified from 1 μg of intact total RNA using oligodT beads (TruSeq RNA Sample Preparation Kit, Illumina, USA). The purified mRNA was fragmented at an elevated temperature in the presence of divalent cations, and reverse transcribed with Superscript II Reverse transcriptase (Invitrogen, USA) by priming with random hexamers. Second strand cDNA was synthesized in the presence of DNA polymerase I and RNase H enzymes. The cDNA was cleaned up using Agencourt Ampure XP Solid Phase Reversible Immobilization beads (Beckman Coulter, Switzerland). Illumina Adapters were ligated to the cDNA molecules after end repair and addition of A base. Solid Phase Reversible Immobilization cleanup was performed after ligation. The library was amplified using PCR for enrichment of adapter ligated fragments. The prepared libraries were quantified by a Nanodrop instrument and validated for quality by running an aliquot on High Sensitivity Bioanalyzer Chip (Agilent Technologies, California, USA).

The DNA obtained from the prepared libraries was denatured and sequenced by the Illumina Genome Analyzer IIX, using the sequencing by synthesis method to read 72 bases PE. The raw sequencing data were then extracted from the server using the proprietary Illumina software to obtain Fastq format. Quality check (QC) of raw data was performed using SeqQC-V2.0 program (NGS data QC).

### *De novo* transcriptome assembly and differentially expressed genes

The following methodology for transcriptome assembly was used for each of the pooled samples of the FAZ and the LAZ. Raw reads were assembled using the Velvet-1.1.05 software and transcripts were generated using Oases assembler (Schulz et al., 2012). The transcripts were then subjected to Basic Local Alignment Search Tool (BLAST) analysis against *S. lycopersicum* mRNA and protein sequences from the International Tomato Annotation Group 2 (ftp://ftp.sgn.cornell.edu/genomes/Solanum_lycopersicum/annotation/ITAG2.3_release/). The transcripts with more than 50% identity and 70% query coverage were used for further analysis. Raw reads of the FAZ and the LAZ were aligned to reference cDNA using the Bowtie-0.12.7 program (Langmead et al., 2009). Expression studies were performed based on enrichment calculation of each gene. Enrichment = average read depth × coverage. Genes were considered to be upregulated, down-regulated, or neutral based on the LAZ/FAZ enrichment ratio. The parameters definitions were as follows: Up, log fold change >1; Down, log fold change <-1; Neutral, log fold change >-1 to <1.

### Design of the AZ-specific microarray (pooled data of the FAZ and the LAZ)

The following steps were performed: Raw reads of both the FAZ and the LAZ samples were pooled and assembled as described above for *de novo* assembly (steps 1 and 2); Unannotated transcripts were subjected to BLAST analysis against *Arabidopsis thaliana* and *Nicotiana tabacum* mRNA and protein sequences, with references derived from The National Center for Biotechnology Information (NCBI) and The Arabidopsis Information Resource (TAIR) databases (step 3); Transcripts with more than 50% identity and 70% query coverage were considered as annotated, and all the rest were categorized as novel transcripts (step 4); Pooled reads were further aligned to mRNA sequences of *S. lycopersicum* and unaligned reads were assembled to derive contigs. These contigs were further filtered by *E. coli, Cestrum elegans, A. thaliana*, and *N. tabacum* mRNA sequences, with references derived from the NCBI database (step 5); Unannotated contigs from the above analysis (step 5) were added to the novel transcripts category (step 6); The unassembled reads from step 5 were further filtered by aligning them to *E. coli* and *C. elegans* mRNA sequences. Unaligned reads were assembled and added to the category of novel transcripts (step 7); Transcripts from steps 4, 6, and 7 were pooled together as novel transcripts, and transcripts which were annotated by *S. lycopersicum, A. thaliana*, and *N. tabacum* as performed in steps 2 and 3 were considered as known transcripts (step 8); To further filter the novel transcripts, BLAST was performed between novel and known transcripts, and only transcripts showing <10% query coverage with known transcripts were considered as novel transcripts (step 9). The novel (step 9) and known (step 8) transcripts were used for the design of the AZ-specific microarray. Probes were categorized as specific and cross hybridizing on the basis of their BLAST results. The criteria for a specific probe were as follows: a probe with a single hit against the target, a probe alignment length of 60–31 bp, a probe with allowed mismatches <3 and gaps <2, and a probe with a minimum length of 28 bases. Thus, out of a total number of 176,026 designed probes, 88,445 were specific probes, and 5363 were cross hybridized probes. For 429 transcripts, no probes were designed as filtered due to repeats and vector masked.

### Gene ontology (GO) term enrichment and biological pathways

Distribution of transcripts into various biological pathways in Kyoto Encyclopedia of Genes and Genomes (KEGG) was done through the KEGG Automatic Annotation Server (http://www.genome.jp/tools/kaas/) to obtain the KEGG IDs for the transcriptome sequences, and to identify the genes involved in plant hormone signal transduction.

### Validation of gene expression by real time PCR (qPCR)

Primers for qPCR analysis were designed manually using the Gene Runner V 3.05 software (Hastings Software Inc. Hastings, USA; http://www.generunner.net). The primers were validated using one of the samples, and the amplicon sizes were confirmed on a 2% agarose gel. The sequences, amplicon length, and melting temperature (Tm) of the primers used are detailed in Table S1. The RNA samples used for the qPCR assay were the same samples used for the microarray analysis. Samples of 400 ng of DNase-treated RNA were reverse transcribed to synthesize 20 ng/μl of cDNA, using an oligo (dT) primer with Affinity Script QPCR cDNA synthesis kit (Agilent Technologies, USA). Relative quantification by qPCR was then performed using Brilliant II SYBR Green qPCR Master mix (Agilent Technologies, USA) according to the manufacturer's instructions. The experiment was conducted using a Stratagene Mx3005P QPCR machine platform (Mx3005P system software, Stratagene, CA). The relative expression levels of the genes were determined after normalizing with *ACTIN* as the reference gene, using the Delta Ct method. The PCR program consisted of an initial denaturation at 95°C for 10 min, followed by 40 cycles of 95°C for 30 s, 60°C for 1 min, and 72°C for 1 min. A melt curve was also performed after the assay to check for specificity of the reaction. Duplicates of each gene with 20 ng cDNA input per reaction from two independent experiments were used. The relative quantification of gene expression level was determined by the comparative C_T_ method 2^−ΔΔC^T.

## Results

### Kinetics of flower pedicel and leaf petiole abscission

The abscission of flower pedicels and leaf petioles was induced by removing the auxin sources, flowers or leaves, respectively. The selected two sets of time points analyzed, 0, 2, 4, 8, and 14 h for flower pedicels and 0, 24, 48, 72, and 96 h for leaf petioles, were based on the abscission kinetics of these organs after flower removal or leaf deblading followed by ethylene treatment, respectively. Flower pedicels completely abscised 14 h after flower removal (Figure 1A), while leaf petioles completely abscised only 96 h after leaf deblading and exposure to ethylene (Figure 1B). The exposure of the leaf debladed explants to ethylene was required to enhance the abscission process, since the petioles abscised very slowly (8–12 days) without ethylene treatment, with a large variation between replicates (data not shown). It should be noted that a similar percentage of organ abscission was obtained in both systems, but on a different time scale (Figure 1). Based on these similar abscission percentages, we have chosen the indicated time points for RNA sampling from both AZs for the RNA-Seq experiments, which correspond to steps 2–4 of the abscission process, namely acquisition of the competence of AZ cells to respond to abscission signals, execution of organ abscission, and beginning of formation of a protective layer. The kinetics of tomato flower pedicel abscission in response to flower removal, without or with exogenous application of ethylene, is well documented (Meir et al., 2010; Wang et al., 2013).

### Illumina paired-end sequencing and *de novo* assembly

The RNA extracts of the FAZ and the LAZ sampled at specified time points after flower or leaf blade removal, respectively, were prepared as described in Figure 1, and sequenced using the Illumina sequencing platform. We used a pooled sequencing strategy by mixing RNA samples of the FAZ and the LAZ from various abscission stages. Individual cDNA libraries for *de novo* transcriptome sequencing were constructed for these pooled samples, in order to obtain a wide range of expressed transcript sequences and to reduce the sequencing costs (Sangwan et al., 2013; Xu et al., 2013). By PE sequencing of the tomato AZs in separate lanes, we obtained a total of 84,626,974 and 80,837,288 reads with 73 bp in length encompassing about 9.05 GB and 9.47 GB of sequence data for the LAZ and the FAZ, respectively (Table 1), in Fastq format after the initial quality filtering was performed with the default parameters. High quality reads were obtained by trimming primers/adapters and filtering by stringent parameters to increase the analysis reliability by trimming the reads with more than 70% of the bases having Phred quality score >20. This resulted in an average of 79,570,559 and 75,843,448 PE reads, which represent 94.02 and 93.82% of high quality read percentage for the LAZ and the FAZ, respectively (Table 1). In addition, this procedure enhanced the average quality score at each base position of the sequence reads by 94.61 and 94.71% (Table S2) of high quality base percentage for the FAZ and the LAZ, respectively (Figures S1, S2).

**Table 1:** **Assembly statistics of pooled transcriptome of the tomato LAZ and FAZ**.

	**LAZ**	**FAZ**
**SEQUENCING**		
Total number of reads	84,626,974	80,837,288
Total number of high quality reads	79,570,559 (94.02%)	75,843,448 (93.82%)
Mean read length (bp)	73	73
Number of reads assembled	65,043,974	61,796,756
Percentage of reads assembled	76.8596	76.4459
**CONTIGS**		
K-mer length used	47	51
Number of generated contigs	25,046	26,583
Maximum contig length (bp)	12,969	9756
Minimum contig length (bp)	100	101
Average contig length (bp)	1048.62	858.539
Total contigs length (bp)	26,263,627	22,822,531
Total number of non-ATGC characters	3784	6364
Percentage of non-ATGC characters	0.000144	0.000279
Number of contigs > 100 bp	25,005	26,583
Number of contigs > 500 bp	16,164	14,132
Number of contigs > 1 Kbp	10,451	8860
Number of contigs > 10 Kbp	3	0
Number of contigs > 1 Mbp	0	0
n50_Log	1581	1512

Initially, we performed a *de novo* assembly for the transcripts, and later on the reference-based assembly was performed using the tomato genome sequence (The Tomato Genome Consortium, 2012) as reference genome. Both the *de novo* and reference assemblies were merged to generate the final assembly, which was used for all further analyses. We also carried out an analysis of differentially expressed genes (DEG) for the FAZ and the LAZ. *De novo* assembly was optimized using different criteria, such as the numbers of used reads, total number of contigs, contigs length in bp (>100, 500, 1000 bp and so on), n50_Log, maximum, average, and minimum contigs length (100 bp), based on the function of K-mer length (Hash Length). The inversed relation between the k-mer and the number of contigs (Sangwan et al., 2013) led us to optimize the assembly with various k-mer values ranging from 33 to 65 (Table S2). At K-mer = 33 the highest number of contigs, 49,849 and 57,398, and a higher number of longer coting length (>10 Kb) was obtained in both the FAZ and the LAZ samples, respectively (Table S2). We assembled most of the high quality reads, 78.74 and 78.41%, into longer contigs at k-mer = 39 and 37 in the FAZ and the LAZ samples, respectively. These recorded the highest percentage of assembled reads (Table 1 and Table S2), thereby implying a high coverage for these sequencing data. The generated assembly had total sequences of 33,025 and 41,592, with an average sequence length of ~1233 and 1255 bp, and a minimum sequence length of 100 bp in the FAZ and the LAZ samples, respectively. Ultimately, FASTA files containing the assembled contigs were obtained for the FAZ and the LAZ, respectively. Table S2 presents a general view of the sequencing and assembly processes, which provides the length distribution for these high-quality reads. The statistics for the two assemblies is detailed in Table 1.

### Differentially expressed gene analysis for the FAZ and the LAZ

**Figure 2:**
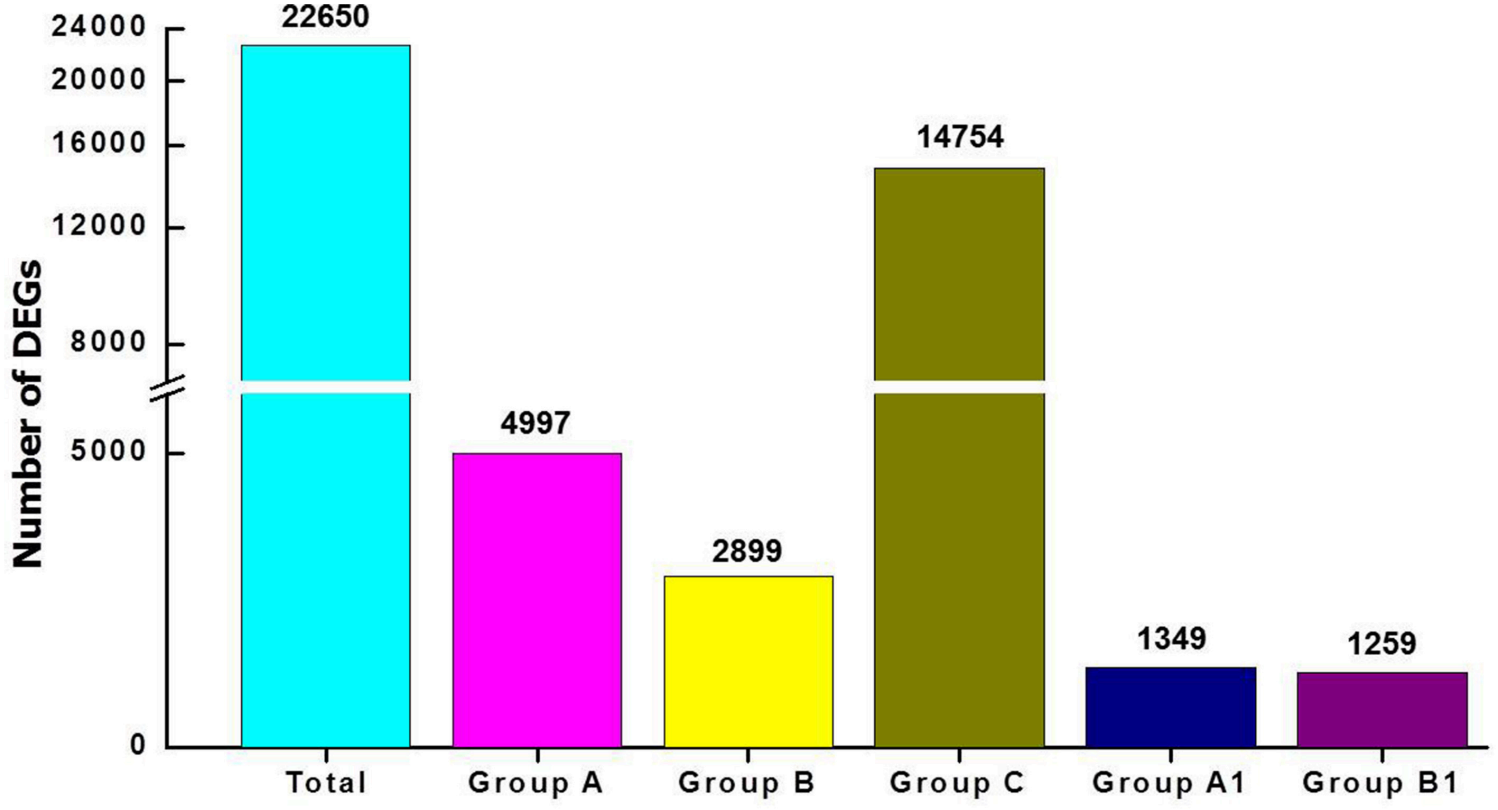
**Analysis of differentially expressed genes (DEG) in pooled samples of FAZ and LAZ**. The number of transcripts indicated above the bars was obtained after performing the Illumina DEG analysis and dividing into groups. Total, transcripts expressed in both tissues regardless of the expression pattern; Group A, transcripts over-expressed in LAZ samples; Group B, transcripts over-expressed in FAZ samples; Group C, transcripts equally expressed in FAZ and LAZ samples; Group A1, transcripts exclusively expressed only in LAZ samples; Group B1, transcripts exclusively expressed only in FAZ samples. The detailed lists of genes in each category are summarized in Table S2.

To investigate abscission distinctions, we compared the transcriptomes of tomato FAZ and LAZ using Illumina DEG analysis. Specifically, we analyzed variations in gene expression between the FAZ and the LAZ in pooled samples, which resulted from two independent DEG libraries. Raw reads of the FAZ and the LAZ were aligned to reference cDNA using the Bowtie-0.12.7 program. The expression study was performed based on enrichment calculation of each gene, when enrichment = average read depth × coverage. The total contigs were classified into five groups, based on their differentially expression levels in the pooled LAZ and FAZ samples, expressed by the LAZ/FAZ enrichment ratio, as follows: Group A, contigs over-expressed in the LAZ; Group B, contigs over-expressed in the FAZ; Group C, contigs equally expressed in the FAZ and the LAZ; Group A1, contigs exclusively expressed in the LAZ; Group B1, contigs exclusively expressed in the FAZ. The parameters used for this classification included: Group A, genes which were up-regulated in the LAZ samples compared to FAZ samples with log fold change >1; Group B, genes which were down-regulated in the LAZ samples compared to the FAZ samples, with log fold change <-1; Group C, genes which were equally expressed in both AZs with log fold change ranging between −1 to +1. The results presented in Figure 2 show that out of 22,650 total genes, 4997 genes were classified in Group A, 2899 genes in Group B, 14,754 genes in Group C, 1349 genes in Group A1, and 1259 genes in Group B1. The number of genes expressed in the LAZ pooled sample was approximately twice the number of genes expressed in the FAZ pooled sample. Table S3 presents the detailed lists of genes expressed in each category.

### Annotation and gene ontology functional enrichment analysis

The tomato genome (The Tomato Genome Consortium, 2012) was recently published, and the functional annotation and assignment of GO by the ITAG enabled us to clearly designate the differences in gene expression between the two tomato AZs. However, the complete transcriptome information on the tomato FAZ and LAZ has not yet been determined. Annotation of assembled genes was performed using the Velvet-1.1.05 and Oases assembler programs and subjected to BLAST analysis against the *S. lycopersicum* mRNA and protein sequences from ITAG2.3 (ftp://ftp.sgn.cornell.edu/genomes/Solanum_lycopersicum/annotation/ITAG2.3_release/). Transcripts showing more than 50% identity and 70% query coverage were taken for further analysis. The average sequence length was 11,840 and 15,794 bp for the FAZ and the LAZ, respectively, with a minimum sequence length of 100 bp. The annotation description of mRNA and proteins is detailed in Table 2. The results indicate that out of 41,592 and 33,025 total contigs generated for the LAZ and the FAZ, respectively, 41 and 49% compared well with the annotated genes and proteins of tomato in the LAZ and the FAZ, respectively. On the other hand, we found that ~58.9 and 50.5% contigs of the LAZ and the FAZ, respectively, failed to map to ITAG2.3 identities of both mRNA and proteins (Table 2). This pool may serve as a good source for discovering new genes. The lists of mRNA-annotated, protein-annotated, mRNA-overlapping, and protein-overlapping contigs are detailed in Tables S4, S5. Once again the assembly was annotated with the recent available genome assembly (The Tomato Genome Consortium, 2012), which resulted in 32,949 and 41,502 transcripts (FASTA file) for the FAZ and the LAZ, respectively (data not shown).

**Table 2:** **Summary of the modules for contig annotation in the LAZ and FAZ**.

**Contig annotation summary**	**LAZ**			**FAZ**		
	**mRNA and proteins**	**mRNA**	**Proteins**	**mRNA and proteins**	**mRNA**	**Proteins**
Total number of contigs	41,592	41,592	41,592	33,025	33,025	33,025
Total number of annotated contigs	17,088	15,232	15,741	16,343	14,717	14,773
Total number of unannotated contigs	24,504	26,360	25,851	16,682	18,308	18,252
Number of overlapping contigs in *Solanum lycopersicum*						
Number of mRNA showing annotation for more than one contig		4052			3988	
Number of proteins showing annotation for more than one contig			9272			9067

**Figure 3:**
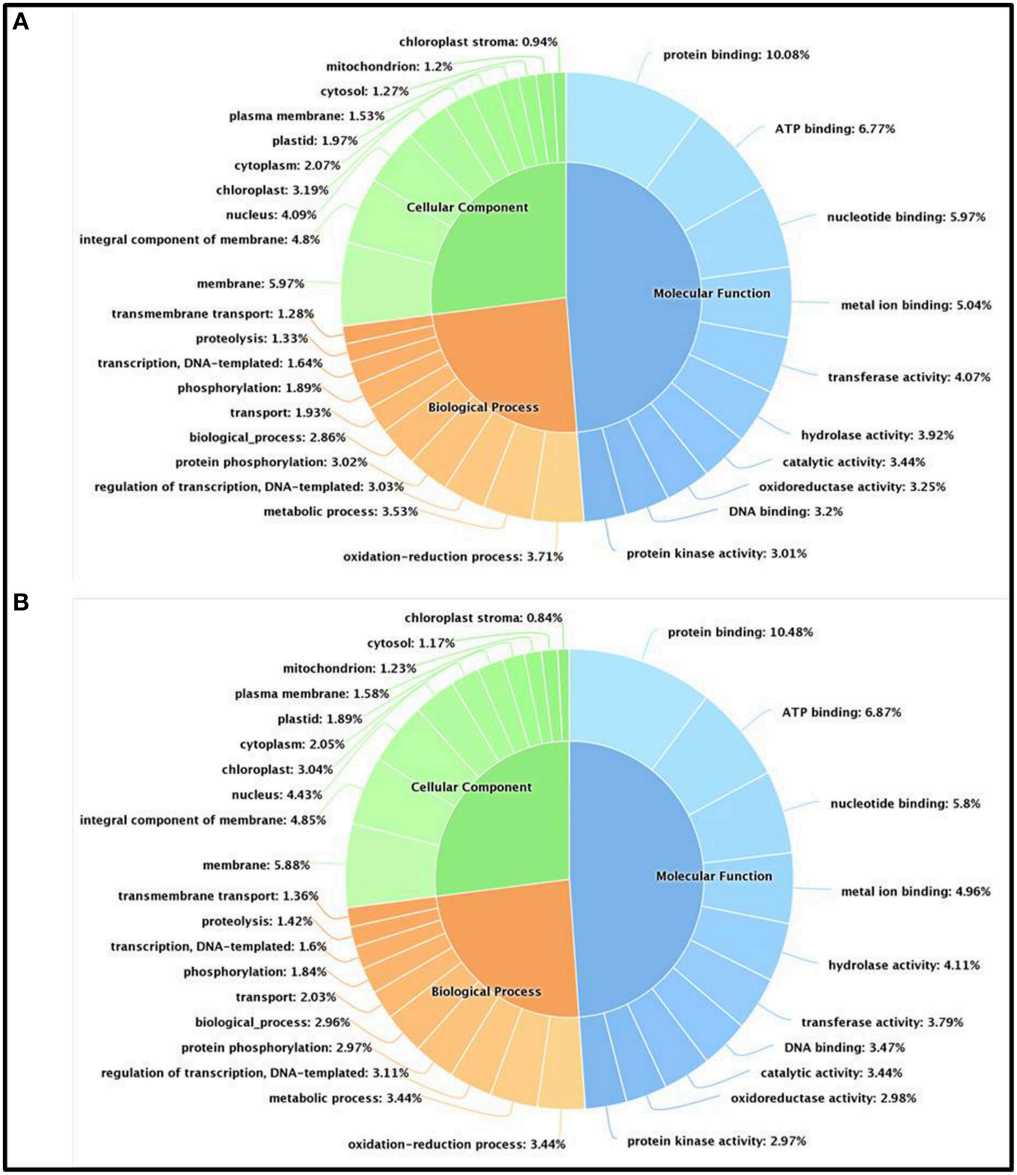
**Enrichment Gene Ontology (GO) terms in the FAZ (A) and LAZ (B)**. The enrichment analysis included 14,773 genes for the FAZ and 15,741 genes for the LAZ. The chart represents the top 10 GO listed in Tables S5, S6 in the categories of Molecular Function (blue), Biological Process (orange), and Cellular Components (green).

We have further carried out an enrichment GO analysis for the total annotated proteins (complete list), including 15,741 and 14,773 LAZ and FAZ proteins, respectively (Table 2). Among them, 12,409 FAZ and 13,106 LAZ proteins were designated with at least one GO term (Tables S6, S7). The GO terms of “protein binding,” “oxidation-reduction process,” and “membrane” were the most represented ones among the categories of molecular function, biological process, and cellular component, respectively, in both AZs (Figure 3). The analysis represents the top 10 GO in molecular function, biological process, and cellular component for the FAZ (Figure 3A) and the LAZ (Figure 3B). These data show that both the FAZ and the LAZ share a similar type of gene enrichments when samples of all-time points following abscission induction were pooled together.

**Figure 4:**
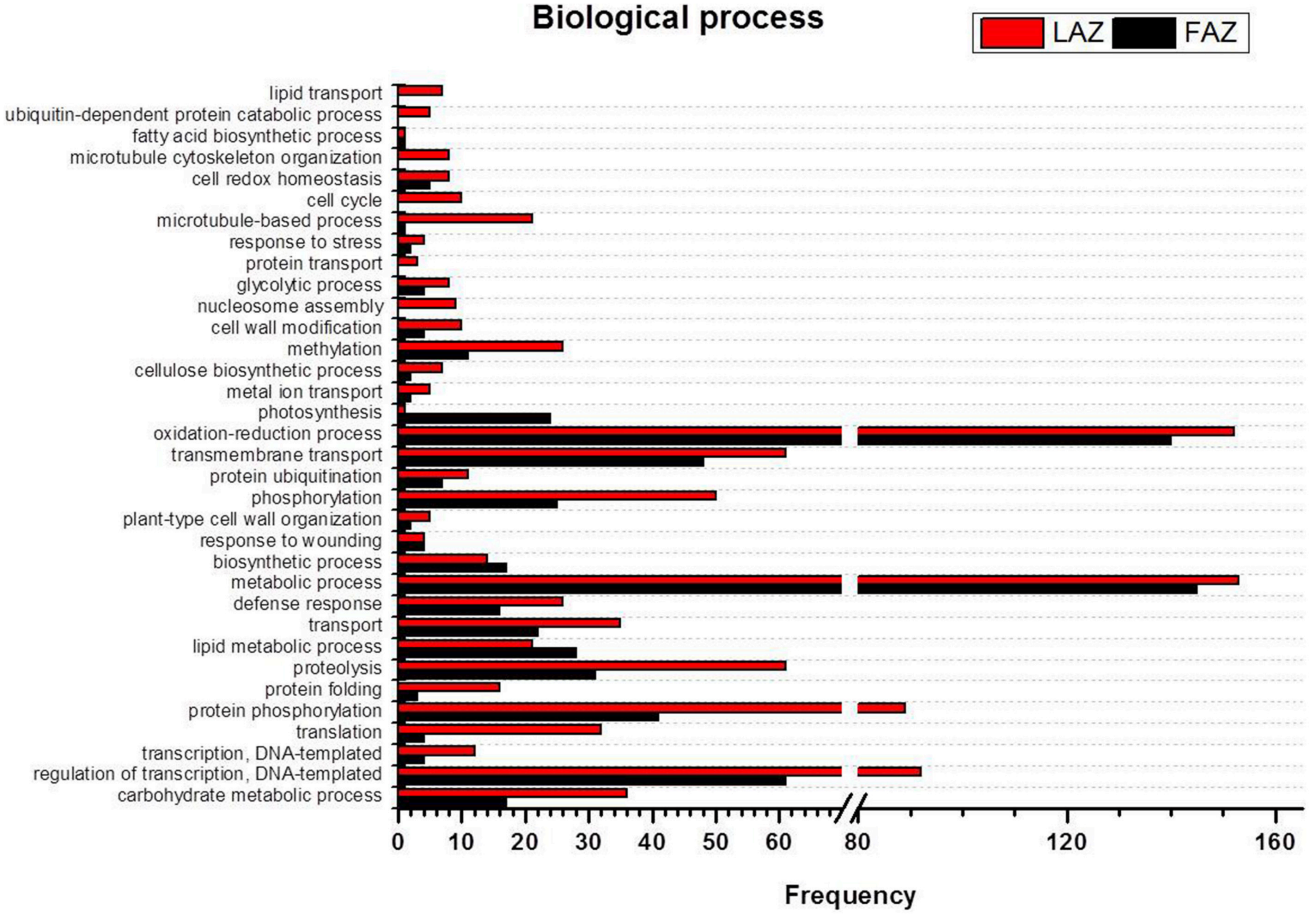
**Comparison of frequencies of the GO “biological process” term of over-expressed transcripts in the LAZ (Group A) and FAZ (Group B)**. The bars represent the comparison of the occurrence frequencies of the GO “biological process” term in the GO annotations of 1135 and 2066 over-expressed transcripts in the FAZ and LAZ, respectively. The frequencies are given for the most abundant biological processes.

**Figure 5:**
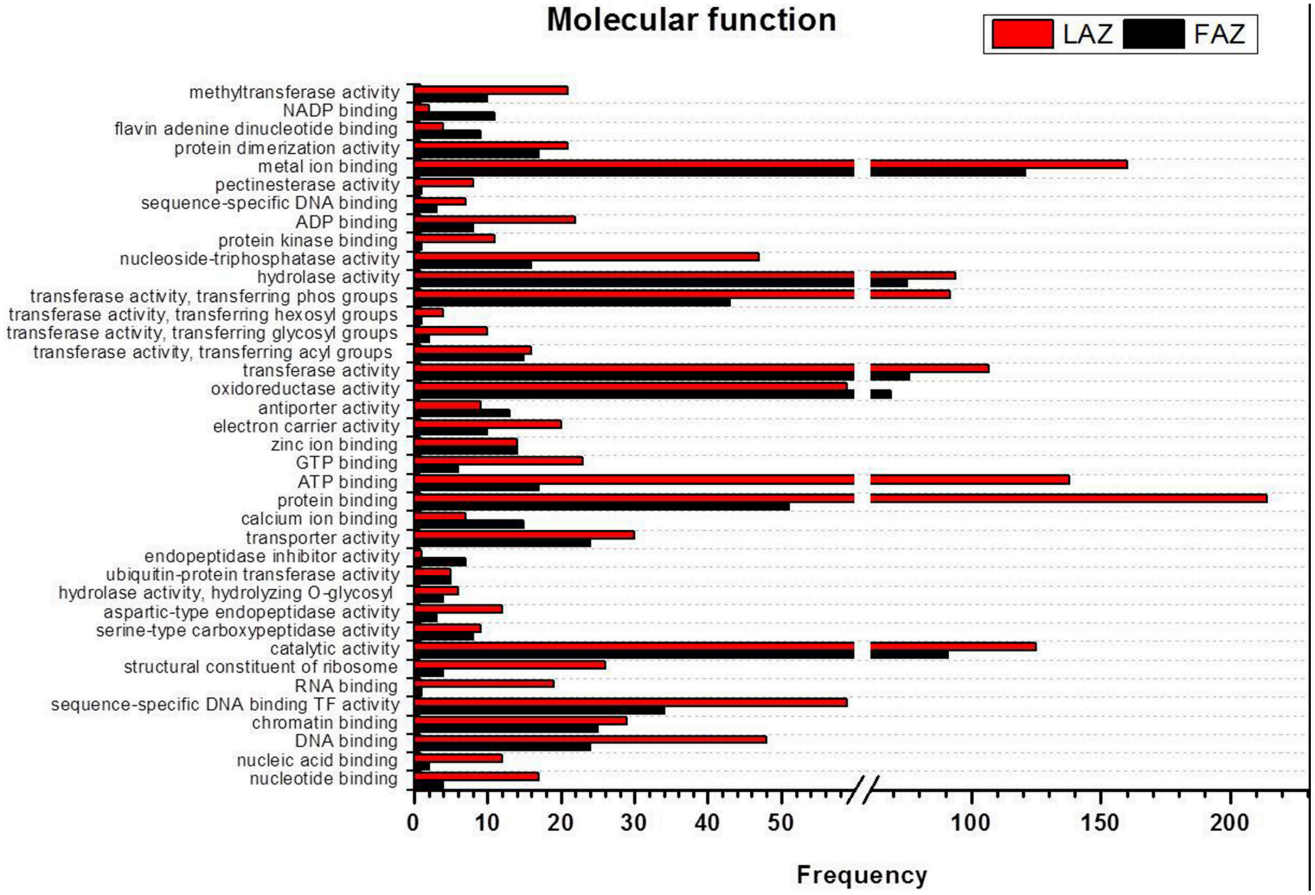
**Comparison of frequencies of the GO “molecular function” term of over-expressed transcripts in the LAZ (Group A) and FAZ (Group B)**. The bars represent the comparison of the occurrence frequencies of the GO “molecular function” term in the GO annotations of 1135 and 2066 over-expressed transcripts in the FAZ and LAZ, respectively. The frequencies are given for the most abundant molecular functions.

**Figure 6:**
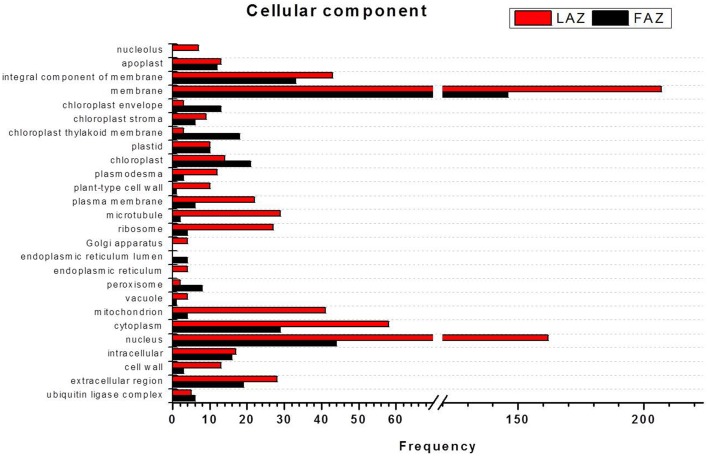
**Comparison of frequencies of the GO “cellular component” term of over-expressed transcripts in the LAZ (Group A) and FAZ (Group B)**. The bars represent the comparison of the occurrence frequencies of the GO “cellular component” term in the GO annotations of 1135 and 2066 over-expressed transcripts in the FAZ and LAZ, respectively. The frequencies are given for the most abundant cellular components.

In the search of cues for explanation of the different abscission rates of petioles and pedicels, we examined the overexpression categories (Groups A and B) in both AZs, as the complete list resulted in similar types of gene enrichments (Figure 3). Out of 4997 and 2899 annotated contigs found in Groups A and B, respectively, 2066 and 1135 were designated with at least one GO term (Tables S8, S9) in the LAZ and the FAZ, respectively. The GO terms identified in Groups A and B (Figures 4–**6**) showed the enrichment of GO categories of highly represented contigs, as demonstrated in the complete annotation charts for both AZs (Figure 3).

The GO terms include indicators of the various biological processes operating in the two AZs during abscission. In the category of biological process, most of the DEG in Group B over-expressed in the FAZ were classified as associated with metabolic processes, oxidation-reduction, regulation of transcription, transmembrane transport, protein amino acid phosphorylation, and proteolyis. Interestingly, Group A (over-expressed in the LAZ) also showed enrichment in a similar list of genes (Figure 4), indicating that the same biological processes might require the operation of the same gene sets in the two AZs during abscission execution. Nevertheless, some differences were found between the two lists of enriched GO terms. In Group A, the GO terms were associated with phosphorylation, transport, carbohydrate metabolic processes, translation, methylation, defense response, microtubule-based processes, protein folding, and cell cycle (Figure 4). This suggests that such biological processes may be associated with leaf abscission. On the other hand, in Group B the enriched GO terms included the processes of lipid metabolism, photosynthesis, and biosynthesis (Tables S8, S9).

In the category of molecular function, the abundant transcripts in Groups A and B showed the predominant expression of genes associated with metal-ion binding, catalytic, transferase, hydrolase activities, and transferring phosphorus-containing groups. Apart of these similar gene categories, the most over-represented GO terms in Group A also included genes associated with protein and ATP binding, while Group B also included genes associated with oxidoreductase activity (Figure 5, Tables S8, S9). Finally, within the category of cellular component, the GO terms of membrane, nucleus, cytoplasm, and integral to membrane constituted the most over-represented categories of the genes with increased transcript accumulation in both Groups A and B (Figure 6).

### Differential regulation of genes encoding transcription factors in the FAZ and the LAZ

**Figure 7:**
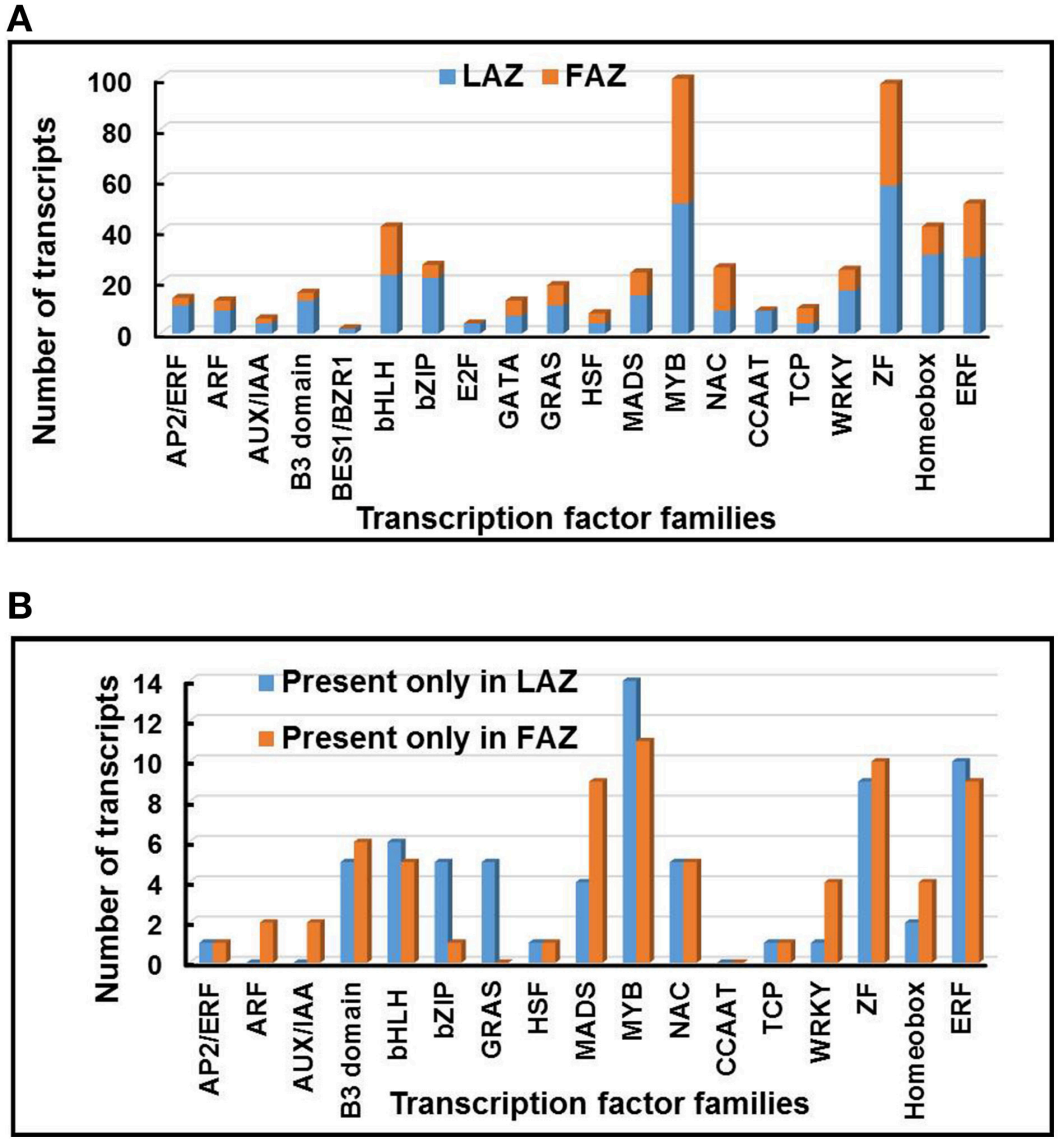
**Distribution of abscission-regulated transcription factor (TF) families over-expressed in the LAZ or in the FAZ (A), and exclusively expressed only in the LAZ or FAZ (B) during abscission**. The changes in the abundance of 551 TF transcripts belonging to 20 families were determined in graph **(A)** for Group A (LAZ) and Group B (FAZ). The changes in the abundance of 141 TF transcripts belonging to 20 families were determined in graph **(B)** for Group A1 (LAZ) and Group B1 (FAZ). The Groups were classified according to the categories presented in Figure 2.

Among the total 7896 differentially regulated genes of Groups A and B, diverse families of genes putatively encoding transcription factors (TFs) were differentially expressed (Table S10) in the pooled samples of the LAZ and the FAZ. Thus, 336 TF genes were expressed in Group A and 215 TF genes were expressed in Group B. Changes in the abundance of these 551 differentially over-expressed TF genes, belonging to the top 20 highly represented TF families, were determined in both the LAZ and the FAZ (Figure 7A). Additionally, diverse families of TF genes were also equally expressed in the FAZ and the LAZ (Group C). Apart from the differentially overexpressed TF genes, we also found exclusively expressed TF genes in each of the AZs. Thus, 71 and 70 TF genes, which belong to various TF families, were specifically expressed in the LAZ and the FAZ, respectively (Group A1, B1) (Table S10, Figure 7B).

The most abundant TF genes overexpressed in the LAZ (Group A) were: ZINC FINGER (ZF) proteins, MYB, Homeobox domain proteins, Ethylene Responsive Factor (ERF), basic leucine zipper (bZIP), basic helix–loop–helix (bHLH), and WRKY. In the FAZ (Group B) the most abundant TF genes were: MYB, ZF proteins, ERF, bHLH, NAC, Homeobox domain proteins, and MADS box (Figure 7A). The most abundant TF genes found in the exclusively expressed genes in the LAZ (Group A1) included: MYB, ERF, ZF proteins, bHLH, GRAS, bZIP, and B3 domain families, whereas the exclusively expressed genes in the FAZ (Group B1) was enriched with the TF genes of MYB, ZF proteins, ERF, MADS box, and B3 domain (Figure 7B). Although the two sub-groups (A1, B1) contained members of several TF families, in each primary Group (A, B), significant differences were found in the proportion of the TF families. Moreover, distinct TF families were differentially expressed in each Group, including AP2/ERF, Auxin Response Factor (ARF), Aux/IAA, TF E2F, and CCAAT-binding protein families in Group A, and NAC, TCP, GATA TF—Zinc finger GATA-type, and GRAS families in Group B (Figure 7A). The enrichment of sequence elements in different gene groups from each cluster, in combination with the data on transcript abundance offer a tenable set of TFs which bind these elements, and that could be examined in future research.

Our results show that the most abundant TF family in Groups A and A1 or Groups B and B1 was MYB (Figure 7). The *MYB* gene (Solyc11g069030—*Bl*) was specifically expressed in the tomato FAZ compared to proximal and distal NAZ tissues (Nakano et al., 2013), and was overexpressed in the LAZ (Group A) as shown by our analysis (Table S10). Several MYB *blind-like* (*bli*) genes (*bli1,3,4,5,7*) (Solyc09g008250, Solyc04g077260, Solyc12g008670, Solyc08g065910, Solyc02g091980, respectively) were also expressed in all groups (Table S10). Our analysis show that a total number of 98 ZF TF genes were overexpressed in both AZs, being the second most highly expressed family in Groups A and B (Figure 7A, Table S10).

The MADS-box and GRAS TFs gene families were expressed in both AZs represented by Groups A and B (Figure 7A, Table S10). While the GRAS family was highly expressed in Group A1, the MADS-box family was much more highly expressed in Group B1 (Figure 7B, Table S10). The MADS-box and GRAS TF genes, *MACROCALYX* (Solyc05g012020), *JOINTLESS* (Solyc11g010570)*, SEPALLATA MADS-box Protein21 (SLMBP21)*, and *Ls* (Solyc07g066250), which were shown to regulate the differentaion and development of the tomato FAZ (Schumacher et al., 1999; Mao et al., 2000; Nakano et al., 2012; Liu et al., 2014), were expressed in both the FAZ and the LAZ (Table S10). The *MACROCALYX* gene was highly expressed in the tomato FAZ (Group B), whereas the *JOINTLESS* gene was expressed at a similar level in both the FAZ and the LAZ (Group C) (Table S10). Therefore, we speculate that a similar type of organ identity specification, reported for the FAZ, might also operate in the LAZ.

The MADS-box gene *Tomato AGAMOUS-LIKE12* (*TAGL12*), which is known to be expressed during tomato seed and fruit development (Busi et al., 2003), was upregulated in the FAZ after flower removal (Meir et al., 2010). Our analysis data show that *TAGL12* was equally present in both the FAZ and the LAZ (Group C), while *TAGL2* (Solyc02g089200—*LeSEP1*) was highly over-expressed in the FAZ (Group B) compared to the LAZ (Group A) (Table S10), in accordance with our previous report (Meir et al., 2010). Several GRAS TFs, including *GRAS2* (Solyc07g063940), *GRAS5* (Solyc09g018460), *GRAS7* (Solyc07g065270), and *GRAS9* (Solyc06g036170), were exclusively over-expressed in the FAZ (Group B), whereas *GRAS4* (Solyc01g100200) was overexpressed in the LAZ (Group A) (Table S10). This suggests that different GRAS TF family members probably mediate abscission-responsive transcription in both flowers and leaves.

The WRKY TF family identified in multiple crop species, was implicated to operate in various biological processes in plants, especially in regulating defense mechanisms against biotic and abiotic stresses (Rushton et al., 2010). So far, 137, 89, and 81 *WRKY* genes were identified in rice, Arabidopsis, and tomato, respectively (Zhang et al., 2011; Huang et al., 2012). Our analyses revealed that 17 and 8 *WRKY* genes were overexpressed in the tomato LAZ and FAZ, respectively (Table S10). These results are consistent with previous studies showing upregulation of several *WRKY* TF genes in fruit AZ during abscission of mature melon and olive fruit (Corbacho et al., 2013; Gil-Amado and Gomez-Jimenez, 2013). *WRKY1* and *WRKYIId-1* (AY157063) genes were upregulated in the tomato FAZ at the early and late stages of pedicel abscission (Meir et al., 2010). In the present study, we show that the *WRKYII* (Solyc01g079360) gene was expressed in both tomato AZs, but was highly expressed in the FAZ (Group B) compared to the LAZ (Table S10). This suggests that the *WRKY* TFs have a role in both AZs in mediating the late events of the abscission process.

Recently, 159 and 152 *bHLH* TFs genes were identified in the tomato genome (Sun et al., 2015; Wang et al., 2015). Out of the 159 identified *bHLH* genes in tomato, we could detect 137 *bHLH* TFs in both AZs, which were distributed among all the groups presented in Figure 2, indicating that this TF family is associated with the abscission process. Out of these 137 AZ-associated *bHLH* genes, 30 genes were expressed in Group A, 28 genes in Group B, 67 genes in Group C, and 7 and 5 genes were exclusively present in the LAZ (Group A1) and the FAZ (Group B1), respectively (Figure 7, Table S10). Most of the *bHLH* TFs were overexpressed in both the FAZ and the LAZ (Group A and B) (Table S10).

Most of the *bZIP* TFs genes (22) were present and ove-expressed in the LAZ (Group A), compared to only five genes in the FAZ (Group B) (Figure 7A, Table S10). In the exclusively expressed categories, five *bZIP* TF genes were present in the LAZ compared to one gene in the FAZ (Figure 7B, Table S10). One of the *bZIP* TF gene (BG631669) was reported to be downregulated in the FAZ at an early stage of tomato pedicel abscission (Meir et al., 2010). Most members of the *AP2/ERF*, B3 domain, *bHLH, bZIP, MADS, MYB, WRKY, ZF, Homeobox*, and *Ethylene Responsive Factor* (*ERF*) gene families were overexpressed in the tomato LAZ samples compared to the FAZ, while most members of the NAC families were less expressed in the LAZ samples (Group A) (Figure 7).

In the present study, we identified common and distinct TFs that were not previously related to abscission. Our results also show that distinct patterns of transcriptional regulation occur in the tomato FAZ and LAZ.

### Key meristem genes in the AZs

A long list of shoot apical meristem (SAM) genes were similarly expressed in both the LAZ and the FAZ (Group C) and in the differentially regulated groups (Group A and B) (Table S3). The key Arabidopsis SAM activity genes and their orthologs were preferentially expressed in tomato FAZ (Nakano et al., 2012; Wang et al., 2013). Our data show that the well-documented key meristem genes, *KNOTTED2* protein/*Tkn3*/*KNAT6* (Solyc05g005090), *BEL1-like homeodomain protein4* (*TBL4*)/*BELL-like homeodomain protein3* (*BLH*) (Solyc08g065420), along with the *LATERAL ORGAN BOUNDARIES DOMAIN PROTEIN1* (*LBD1*) (Solyc11g072470), and the tomato auxiliary meristem gene, *Bl* (Solyc11g069030), which encodes a MYB TF, were highly expressed in the tomato LAZ (Group A) compared to the FAZ (Table 3). On the other hand, the WUSCHEL-related homeobox-containing protein4 (*WUS*) gene (Solyc02g083950), *OVATE FAMILIY PROTEIN* (*OFP*) gene (Solyc02g085500), another *MYB-Cpm10*/*MYB78* gene (Solyc05g053330), *Goblet* (Solyc07g062840), a NAC domain TF gene, and the *Ls* (Solyc07g066250), a GRAS family TF gene, were highly expressed in the tomato FAZ compared to the LAZ (Group B) (Table 3).

**Table 3:** **Differential expression patterns of shoot meristem genes in the tomato LAZ and FAZ**.

**Gene ID**	**Solyc gene description^a^**	**Expression level**		**Log_2_ (Ratio)^b^**
		**LAZ**	**FAZ**	
Solyc08g065420^*^	*BEL1-like homeodomain protein 3 (BLH)/TBL4*	12,966	2564	2.34
Solyc07g066250^*^	*GRAS family TF (LATERAL SUPRESSOR-Ls)*	45	783	−4.11
Solyc05g005090^*^	*Knotted1-like homeobox protein H1 (Knotted2 – KNAT6)/TKn3*	7975	2577	1.63
Solyc02g083950^*^	*WUSCHEL-related homeobox-containing protein4 (WUS)*	36	178	−2.32
Solyc11g069030^*^	*MYB TF (Bl)*	9703	1971	2.30
Solyc11g072470^*^	*LOB domain protein1(LBD1)*	27,817	6660	2.06
Solyc02g085500^*^	*Ovate protein*	1343	4640	−1.79
Solyc05g053330^*^	*MYB TF*	9779	22,747	−1.22
Solyc07g062840	*NAC domain protein (GOBLET)*	647	2715	−2.07

* *Genes which were shown to be preferentially expressed in the tomato FAZ (Meir et al., 2010; Nakano et al., 2013; Wang et al., 2013)*.

a *According to the Tomato Sol Genomic Network database (http://solgenomics.net/)*.

b *Log_2_ of the gene expression ratio between LAZ and FAZ. Ratio = LAZ/FAZ*.

### Cell wall related genes in the FAZ and the LAZ

Our RNA-Seq analyses show that several genes encoding cell wall and middle lamella degradation and remodeling factors, which are the main targets at the late stages of the abscission process, were expressed in both the tomato FAZ and LAZ. These include *PG, Cel, XTH*, and *EXP* genes (Table 4). These genes were previously demonstrated to be highly expressed in the AZs of a large number of abscising organs (Lashbrook et al., 1994; del Campillo and Bennett, 1996; Cho and Cosgrove, 2000; Taylor and Whitelaw, 2001; Roberts et al., 2002; Ogawa et al., 2009; Meir et al., 2010). The previously characterized tomato AZ-specific PG genes (*TAPGs*), *TAPG1,2,3,4,5*, were expressed in both the FAZ and the LAZ, with *TAPG4* expression being the highest and *TAPG3* expression being the lowest in both AZs (Table 4). These results are in agreement with previous works showing that *TAPG1,2,4* were specifically expressed in tomato FAZ (Lashbrook et al., 1994; Kalaitzis et al., 1997; Wang et al., 2013), and their expression was inhibited by 1-methylcyclopropene (1-MCP) in a positive correlation with its inhibitory effect on pedicel abscission (Meir et al., 2010).

**Table 4:** **Differential expression patterns of cell wall-related genes in the tomato LAZ and FAZ**.

**Gene ID**	**Gene name**	**Nucleotide Accession number**	**NCBI description**	**Expression level**		**Log_2_(Ratio)**
				**LAZ**	**FAZ**	
**POLYGALACTURONASES**						
Solyc02g067630	*TAPG1*	AF001000	Polygalacturonase 1	93,779	65,322	0.5
Solyc02g067640	*TAPG2*	AF001001	Polygalacturonase 2	19,182	58,301	−1.6
Solyc02g067650	*TAPG3*	AF000999	Polygalacturonase 3	661	399	0.7
Solyc12g096750	*TAPG4*	AF001002	Polygalacturonase 4	93,458	298,246	−1.7
Solyc12g096740	*TAPG5*	AF001003	Polygalacturonase 5	40,338	169,016	−2.1
Solyc12g019180		AF072732	Polygalacturonase 7	819	37	4.5
Solyc12g096730	*TPG6*	AF029230	Polygalacturonase	258	123	1.1
Solyc03g116500	*XOPG1*	AF138858	Polygalacturonase	8036	2546	1.7
Solyc08g060970	*PGcat*	AF118567	Polygalacturonase	15,150	10,785	0.5
Solyc04g015530	*PS-2*	EU111748	Dehiscence polygalacturonase	0	2774	0.0
Solyc10g080210	*PG-2a*	X04583	Polygalacturonase-2a	0	11	0.0
Solyc07g065090		L26529	Polygalacturonase inhibitor protein	49,824	52,806	−0.1
**CELLULASES**						
Solyc08g081620	*Cel1*	U13054	Endo-1,4-beta-glucanase	7882	27,048	−1.8
Solyc09g010210	*Cel2*	U13055	Endo-1,4-beta-glucanase	33,292	4618	2.8
Solyc01g102580	*Cel3*	U78526	Endo-1,4-beta-glucanase	31,216	37,492	−0.3
Solyc09g075360	*Cel4*	U20590	Endo-1,4-beta-glucanase	3210	3800	−0.2
Solyc08g083210	*Cel5*	AF077339	Endo-1,4-beta-glucanase	256,927	10,075	4.7
Solyc05g005080	*Cel6*	NA	Endo-1,4-beta-glucanase	87,695	30,178	1.5
Solyc11g040340	*Cel7*	Y11268	Endo-1,4-beta-glucanase	14,458	1561	3.2
Solyc08g082250	*Cel8*	AF098292	Endo-1,4-beta-glucanase	47,547	7409	2.7
**XYLOGLUCAN ENDOTRANSGLUCOSYLASE/HYDROLASES**						
Solyc01g099630	*SlXTH1*	D16456	Endo-xyloglucan transferase	299,193	15,747	4.2
Solyc07g009380	*SlXTH2*	AF176776	Xyloglucan endotransglycosylase lexet2	19,890	3315	2.6
Solyc03g093080	*SlXTH3*	AY497476	Xyloglucan endotransglucosylase-hydrolase XTH3	11,463	7902	0.5
Solyc03g093110	*SlXTH3*	AY497476	Xyloglucan endotransglucosylase-hydrolase XTH3	10,544	6556	0.7
Solyc03g093120	*SlXTH3*	AY497476	Xyloglucan endotransglucosylase-hydrolase XTH3	11,633	6461	0.8
Solyc03g093130	*SlXTH3*	AY497476	Xyloglucan endotransglucosylase-hydrolase XTH3	12,099	16,273	−0.4
Solyc11g065600	*SlXTH4*	AF186777	Xyloglucan endotransglycosylase	83	795	−3.3
Solyc01g081060	*SlXTH5*	AY497475	Xyloglucan endotransglucosylase-hydrolase XTH5	32,612	54,930	−0.8
Solyc11g066270	*SlXTH6*	AY497477	xyloglucan endotransglucosylase-hydrolase XTH6	30,396	4588	2.7
Solyc02g091920	*SlXTH7*	AY497478	Xyloglucan endotransglucosylase-hydrolase XTH7	11,361	16,524	−0.5
Solyc04g008210	*SlXTH8*	AB036338	endoxyloglucan transferase	14,512	18,467	−0.3
Solyc12g011030	*SlXTH9*	AY497479	xyloglucan endotransglucosylase-hydrolase XTH9	25,489	12,237	1.1
Solyc07g056000	*SlXTH10*	X82684	Xyloglycan endo-transglycosylase	21,410	28,794	−0.4
Solyc12g017240	*SlXTH11*	X82685	Xyloglucan endo-transglycosylase	6763	3776	0.8
Solyc07g052980	*SlXTH16*	DQ098654	Xyloglucan endotransglucosylase-hydrolase XTH16	43,636	186,375	−2.1
**EXPANSINS**						
Solyc01g090810	*LeEXPB1*	DQ234354	Beta-expansin precursor	2151	0	
Solyc03g093390	*LeEXPB2*	DQ205653	Beta expansin precursor (EXPB2)	102	17	2.6
Solyc05g007830		AC154033	Alpha-expansin 1 precursor	172,124	9441	4.2
Solyc01g112000	*LeEXLA1*	DQ178133	Expansin-like protein precursor (EXLA1)	46,270	23,553	1.0
Solyc06g051800	*LeEXP1*	U82123	Fruit ripening regulated expansin	74,430	3752	4.3
Solyc06g049050	*LeEXP2*	AF096776	Expansin LeEXP2	35,951	10,479	1.8
Solyc03g031840	*EXPA3*	AF059487	Expansin precursor (EXPA3)	18,068	12,366	0.5
Solyc09g010860	*EXPA4*	AF059488	Expansin precursor (EXPA4)	5088	3355	0.6
Solyc02g088100	*EXPA5*	AF059489	Expansin precursor (EXPA5)	67,484	21,494	1.7
Solyc10g086520	*EXPA6*	AF059490	Expansin (EXPA6)	6244	253	4.6
Solyc03g115300	*EXPA7*	AF059491	Expansin	112	75	0.6
Solyc12g089380	*EXPA8*	AF184232	Expansin EXPA8	396	75	2.4
Solyc06g005560	*EXP9*	AJ243340	Expansin9	60,173	13,676	2.1
Solyc03g115890	*EXPA10*	AF184233	Expansin EXPA10	1183	349	1.8
Solyc04g081870	*Exp11*	AF218775	Expansin precursor (Exp11)	81,922	3546	4.5
Solyc06g076220	*Exp18*	AJ004997	Expansin18	99,291	2685	5.2

The expression levels of genes encoding cellulase enzymes, such as *Cel1,2,3,4,5,6,7,8*, are detailed in Table 4. *Cel2,5,6,7,8* were highly expressed in the LAZ (Group A), with *Cel5* showing the highest expression among all the *Cel* family genes in both AZs (Table 4). Only the expression of *Cel1* was higher in the FAZ (Group B) than in the LAZ, while *Cel3* and *Cel4* had similar expression levels in both AZs (Table 4). The expression levels of several *XTH* genes, *SlXTH1,2,6,9*, was higher in the LAZ than in the FAZ samples, while the other genes of this family were similarly expressed in both AZs (Table 4). It was previously reported that *XTH8,9* were highly and specifically expressed in tomato FAZ following ethylene treatment (Wang et al., 2013), and *XTH6* was specifically up-regulated in the tomato FAZ following flower removal, and this increased expression was inhibited by 1-MCP pretreatment (Meir et al., 2010). *XTH1,2* genes were also over-expressed in the LAZ during ethylene-induced citrus leaf abscission (Agustí et al., 2008). These results further confirm the role of ethylene in inducing abscission via increased expression of *XTH* genes in both the LAZ and the FAZ.

In general, all the members of the *EXP* gene family, which were expressed in both tomato AZs, were highly upregulated in the LAZ compared to the FAZ (Table 4). The expression level of several *EXP* genes, *LeEXP1,5,9,11,18*, was particularly high in the LAZ.

### Differential regulation of genes associated with hormonal signal transduction

The RNA-Seq data for both the FAZ and the LAZ samples were analyzed for the KEGG pathway database to examine the potential involvement of consensus sequences in hormonal signal transduction pathways. The pathway-based analysis helped us to understand the biological functions and their interactions (Figures S3, S4). The KEGG categories and list of transcripts in each sample with their expression values in the LAZ and the FAZ are listed in Table 5. The genes related to the different plant hormones, auxin, ethylene, jasmonic acid (JA), abscisic acid (ABA), brassinosteroids, cytokinins, and gibberellins (GA), and other signaling-related factors (protein kinases), were present in both AZs (Table 5). Most of the genes were equally expressed in both AZs (Group C), but some genes of each hormone category were differentially expressed in the LAZ or the FAZ, belonging to Groups A or B, respectively (Figure 2). The data suggest specific roles for this family members in each AZ.

**Table 5:** **The KEGG categories and their expression values for plant hormone signaling-related genes in the tomato LAZ and FAZ**.

**Gene ID**	**KEGG ID**	**KEGG description**	**Expression level**		**Log_2_ (Ratio)**
			**LAZ**	**FAZ**	
**AUXIN**					
Solyc10g076790.1.1	K13946	Auxin influx carrier (AUX1 LAX family) *LAX4*	601	3295	−2.5
Solyc11g013310.1.1	K13946	Auxin influx carrier (AUX1 LAX family) *LAX3*	19,674	29,200	−0.6
Solyc10g055260.1.1	K13946	Auxin influx carrier (AUX1 LAX family) *LAX5*	89	50	0.8
Solyc03g120390.1.1	K14484	Auxin-responsive protein IAA (*IAA8*)	5982	4732	0.3
Solyc03g120500.1.1	K14484	Auxin-responsive protein IAA (*IAA6*)	19,397	60,354	−1.6
Solyc04g076850.1.1	K14484	Auxin-responsive protein IAA (*IAA9*)	50,252	53,992	−0.1
Solyc06g008590.1.1	K14484	Auxin-responsive protein IAA (*IAA10*)	1653	745	1.1
Solyc06g066020.1.1	K14484	Auxin-responsive protein IAA	941	1001	−0.1
Solyc09g083280.1.1	K14484	Auxin-responsive protein IAA (*IAA23*)	9659	2545	1.9
Solyc09g083290.1.1	K14484	Auxin-responsive protein IAA (*IAA24*)	74,088	3881	4.3
Solyc09g090910.1.1	K14484	Auxin-responsive protein IAA (*IAA 25*)	1874	3577	−0.9
Solyc12g007230.1.1	K14484	Auxin-responsive protein IAA (*IAA26*)	19,121	21,111	−0.1
Solyc03g121060.1.1	K14484	Auxin-responsive protein IAA (*IAA14*)	35,233	115,921	−1.7
Solyc08g021820.1.1	K14484	Auxin-responsive protein IAA (*IAA21*)	7129	790	3.2
Solyc09g065850.1.1	K14484	Auxin-responsive protein IAA (*IAA3*)	17,164	13,737	0.3
Solyc01g096070.1.1	K14486	Auxin response factor (*ARF18*)	20,948	4373	2.3
Solyc01g103050.1.1	K14486	Auxin response factor (*ARF1*)	25,076	18,211	0.5
Solyc02g077560.1.1	K14486	Auxin response factor (*ARF3*)	12,141	8367	0.5
Solyc04g081240.1.1	K14486	Auxin response factor (*ARF5*)	2305	2776	−0.3
Solyc07g042260.1.1	K14486	Auxin response factor (*ARF19*)	2634	3400	−0.4
Solyc08g008380.1.1	K14486	Auxin response factor (*ARF9B*)	3429	2597	0.4
Solyc12g005310.1.1	K14487	Auxin responsive GH3 gene family (*GH3-15*)	2800	798	1.8
Solyc01g107390.1.1	K14487	Auxin responsive GH3 gene family (*GH3-2*)	212	2279	−3.4
Solyc10g008520.1.1	K14487	Auxin responsive GH3 gene family (*GH3-10*)	3559	10,863	−1.6
Solyc02g084010.1.1	K14488	SAUR family protein (*SAUR33*)	1843	2835	−0.6
Solyc07g014620.1.1	K14488	SAUR family protein (*SAUR63*)	2031	1128	0.8
Solyc01g091030.1.1	K14488	SAUR family protein (*SAUR1*)	13,748	7391	0.9
Solyc01g110580.1.1	K14488	SAUR family protein (*SAUR5*)	552	151	1.9
Solyc01g110770.1.1	K14488	SAUR family protein (*SAUR10*)	283	63	2.2
Solyc03g082520.1.1	K14488	SAUR family protein (*SAUR36*)	1320	13,445	−3.3
Solyc03g082530.1.1	K14488	SAUR family protein (*SAUR37*)	3442	23,779	−2.8
Solyc02g079190.1.1	K14485	Transport inhibitor response 1	28,232	34,580	−0.3
Solyc09g074520.1.1	K14485	Transport inhibitor response 1	17,058	18,054	−0.1
Solyc03g118740		Auxin efflux carrier (*SlPIN1*)	1559	4048	−1.4
Solyc04g007690		Auxin efflux carrier (*SlPIN3*)	3123	2283	0.5
Solyc05g008060		Auxin efflux carrier (*SlPIN4*)	21,059	23,775	−0.2
Solyc01g068410		Auxin efflux carrier (*SlPIN5*)	1681	163	3.4
Solyc06g059730		Auxin efflux carrier (*SlPIN6*)	290	47	2.6
Solyc10g080880		Auxin efflux carrier (*SlPIN7*)	514	182	1.5
Solyc02g087660		Auxin efflux carrier (*SlPIN8*)	190	218	−0.2
Solyc10g078370		Auxin efflux carrier (*SlPIN9*)	1284	2617	−1.0
**ETHYLENE**					
Solyc01g009170.1.1	K14514	Ethylene-insensitive protein 3 (*LeEIL2*)	53,217	61,035	−0.2
Solyc01g014480.1.1	K14514	Ethylene-insensitive protein 3	5896	5983	0.0
Solyc01g096810.1.1	K14514	Ethylene-insensitive protein 3 (*LeEIL3*)	73,340	97,915	−0.4
Solyc06g073720.1.1	K14514	Ethylene-insensitive protein 3 (*LeEIL1*)	46,978	73,358	−0.6
Solyc06g073730.1.1	K14514	Ethylene-insensitive protein 3 (*LeEIL4*)	47,534	50,923	−0.1
Solyc09g007870.1.1	K14513	Ethylene-insensitive protein 2 (*EIN2*)	22,094	24,356	−0.1
Solyc04g014530.1.1	K14516	Ethylene-responsive transcription factor1 (*ERF.C2*)	2600	2038	0.4
Solyc05g051200.1.1	K14516	Ethylene-responsive transcription factor1 (*ERF.C1*)	25,783	70,599	−1.5
Solyc05g051180.1.1	K14516	Ethylene-responsive transcription factor1	1084	0	0.0
Solyc09g089930.1.1	K14516	Ethylene-responsive transcription factor1 (*ERF.E2*)	5735	9067	−0.7
Solyc11g011740.1.1	K14516	Ethylene-responsive transcription factor1 (*ERF*)	3712	165	4.5
Solyc11g011750.1.1	K14516	Ethylene-responsive transcription factor1	1982	0	0.0
Solyc07g008250.1.1	K14515	EIN3-binding F-box protein	180,332	61,554	1.6
Solyc12g009560.1.1	K14515	EIN3-binding F-box protein	70,361	47,713	0.6
Solyc08g060810.1.1	K14515	EIN3-binding F-box protein	43,384	91,970	−1.1
Solyc06g053710.1.1	K14509	Ethylene receptor [EC:2.7.13.-] (*ETR4*)	39,361	33,454	0.2
Solyc11g006180.1.1	K14509	Ethylene receptor [EC:2.7.13.-] (*ETR5*)	5931	4233	0.5
Solyc12g011330.1.1	K14509	Ethylene receptor [EC:2.7.13.-] (*ETR1*)	7997	9721	−0.3
Solyc09g075440.1.1	K14509	Ethylene receptor [EC:2.7.13.-] (*ETR3*)	41,038	25,627	0.7
**JASMONIC ACID (JA)**					
Solyc10g011660.1.1	K14506	Jasmonic acid-amino synthetase	25,179	79,073	−1.7
Solyc03g118540.1.1	K13464	Jasmonate ZIM domain-containing protein	1637	4703	−1.5
Solyc03g122190.1.1	K13464	Jasmonate ZIM domain-containing protein	6892	147,758	−4.4
Solyc12g009220.1.1	K13464	Jasmonate ZIM domain-containing protein	8060	98,488	−3.6
Solyc01g005440.1.1	K13464	Jasmonate ZIM domain-containing protein	18,902	218,372	−3.5
Solyc11g011030.1.1	K13464	Jasmonate ZIM domain-containing protein	1381	81,182	−5.9
Solyc12g049400.1.1	K13464	Jasmonate ZIM domain-containing protein	5763	189,025	−5.0
Solyc05g052620.1.1	K13463	Coronatine-insensitive protein 1 (*COI1*)	7622	8202	−0.1
**ABSCISIC ACID (ABA)**					
Solyc06g050500.1.1	K14496	Abscisic acid receptor PYR/PYL family	7053	9257	−0.4
Solyc08g076960.1.1	K14496	Abscisic acid receptor PYR/PYL family	6901	4195	0.7
Solyc03g095780.1.1	K14496	Abscisic acid receptor PYR/PYL family	3927	940	2.1
Solyc10g076410.1.1	K14496	Abscisic acid receptor PYR/PYL family	13,049	3674	1.8
Solyc12g095970.1.1	K14496	Abscisic acid receptor PYR/PYL family	26	306	−3.6
Solyc04g078840.1.1	K14432	ABA responsive element binding factor	19,141	13,753	0.5
Solyc10g081350.1.1	K14432	ABA responsive element binding factor	7695	3965	1.0
Solyc01g108080.1.1	K14432	ABA responsive element binding factor	11,528	18,411	−0.7
Solyc09g009490.1.1	K14432	ABA responsive element binding factor	1641	23	6.2
**BRASSINOSTEROID (BA)**					
Solyc01g104970.1.1	K13416	Brassinosteroid insensitive 1-associated receptor kinase 1 [EC:2.7.11.1 2.7.10.1]	14,379	17,613	−0.3
Solyc10g047140.1.1	K13416	Brassinosteroid insensitive 1-associated receptor kinase 1 [EC:2.7.11.1 2.7.10.1]	11,969	12,680	−0.1
Solyc04g039730.1.1	K13416	Brassinosteroid insensitive 1-associated receptor kinase 1 [EC:2.7.11.1 2.7.10.1]	4539	6692	−0.6
Solyc04g051510.1.1	K13415	Protein brassinosteroid insensitive 1 [EC:2.7.11.1 2.7.10.1] (*tBRI1/SR160*)	25,691	15,587	0.7
Solyc01g080880.1.1	K14500	BR-signaling kinase [EC:2.7.11.1]	43,070	28,006	0.6
Solyc10g085000.1.1	K14500	BR-signaling kinase [EC:2.7.11.1]	8438	2869	1.6
Solyc02g072300.1.1	K14502	Protein brassinosteroid insensitive 2 [EC:2.7.11.1]	67,130	52,758	0.3
Solyc07g055200.1.1	K14502	Protein brassinosteroid insensitive 2 [EC:2.7.11.1]	49,911	44,967	0.2
Solyc04g079980.1.1	K14503	Brassinosteroid resistant 1/2	33,117	15,846	1.1
Solyc02g063010.1.1	K14503	Brassinosteroid resistant 1/2	22,858	26,736	−0.2
**PROTEIN KINASES**					
Solyc04g012160.1.1	K14498	Serine/threonine-protein kinase SRK2 [EC:2.7.11.1]	10,971	3460	1.7
Solyc04g074500.1.1	K14498	Serine/threonine-protein kinase SRK2 [EC:2.7.11.1]	11,080	7213	0.6
Solyc09g009090.1.1	K14510	Serine/threonine-protein kinase CTR1 [EC:2.7.11.1] (*CTR3-SlMAPKKK68*)	6144	6104	0.0
Solyc10g083610.1.1	K14510	Serine/threonine-protein kinase CTR1 [EC:2.7.11.1] (*CTR1-SlMAPKKK77*)	10,408	10,150	0.0
Solyc10g085570.1.1	K14510	Serine/threonine-protein kinase CTR1 [EC:2.7.11.1] (*CTR4-SlMAPKKK78*)	3629	5872	−0.7
Solyc12g019460.1.1	K14512	Mitogen-activated protein kinase 6 [EC:2.7.11.24]	23,304	21,882	0.1
Solyc08g014420.1.1	K14512	Mitogen-activated protein kinase 6 [EC:2.7.11.24]	15,807	13,380	0.2
Solyc04g008110.1.1	K14489	Arabidopsis histidine kinase 2/3/4 (cytokinin receptor) [EC:2.7.13.3]	4945	2291	1.1
Solyc05g015610.1.1	K14489	Arabidopsis histidine kinase 2/3/4 (cytokinin receptor) [EC:2.7.13.3]	11,349	20,362	−0.8
Solyc07g047770.1.1	K14489	Arabidopsis histidine kinase 2/3/4 (cytokinin receptor) [EC:2.7.13.3]	4949	4851	0.0
**CYTOKININS**					
Solyc06g048930.1.1	K14492	Two-component response regulator ARR-A family	2613	35,896	−3.8
Solyc01g065540.1.1	K14491	Two-component response regulator ARR-B family	2713	5276	−1.0
Solyc04g008050.1.1	K14491	Two-component response regulator ARR-B family	4064	5417	−0.4
Solyc05g014260.1.1	K14491	Two-component response regulator ARR-B family	11,991	12,016	0.0
Solyc05g054390.1.1	K14491	Two-component response regulator ARR-B family	28,980	22,296	0.4
Solyc07g005140.1.1	K14491	Two-component response regulator ARR-B family	6591	4443	0.6
Solyc12g010330.1.1	K14491	Two-component response regulator ARR-B family	6610	6061	0.1
**GIBBERELLIN (GA)**					
Solyc04g078390.1.1	K14495	F-box protein GID2	44,092	37,565	0.2
Solyc11g011260.1.1	K14494	DELLA protein (*LeGAI*)	27,680	14,393	0.9
**OTHERS**					
Solyc01g102300.1.1	K12126	Phytochrome-interacting factor 3	9224	5635	0.7
Solyc07g043580.1.1	K16189	Phytochrome-interacting factor 4	10,518	6044	0.8
Solyc03g006960.1.1	K14497	Protein phosphatase 2C [EC:3.1.3.16]	1381	1769	−0.4
Solyc03g007230.1.1	K14497	Protein phosphatase 2C [EC:3.1.3.16]	25,140	59,268	−1.2
Solyc03g096670.1.1	K14497	Protein phosphatase 2C [EC:3.1.3.16]	47,447	64,439	−0.4
Solyc03g121880.1.1	K14497	Protein phosphatase 2C [EC:3.1.3.16]	61,668	38,683	0.7
Solyc05g052980.1.1	K14497	Protein phosphatase 2C [EC:3.1.3.16]	44,904	49,082	−0.1
Solyc06g051940.1.1	K14497	Protein phosphatase 2C [EC:3.1.3.16]	4900	1780	1.5
Solyc06g076400.1.1	K14497	Protein phosphatase 2C [EC:3.1.3.16]	6832	9804	−0.5
Solyc07g040990.1.1	K14497	Protein phosphatase 2C [EC:3.1.3.16]	20,065	20,418	0.0
Solyc02g092980.1.1	K14505	Cyclin D3, plant	18,872	2447	2.9
Solyc12g088650.1.1	K14505	Cyclin D3, plant	5434	1717	1.7
Solyc08g076930.1.1	K13422	Transcription factor MYC2	14,494	28,347	−1.0
Solyc07g040690.1.1	K14508	Regulatory protein NPR1	10,172	9185	0.1
Solyc10g079460.1.1	K14508	Regulatory protein NPR1	6214	7924	−0.4
Solyc02g069310.1.1	K14508	Regulatory protein NPR1	9608	10,302	−0.1
Solyc07g044980.1.1	K14508	Regulatory protein NPR1	34,016	38,526	−0.2
Solyc10g079750.1.1	K14508	Regulatory protein NPR1	4588	587	3.0
Solyc04g054320.1.1	K14431	Transcription factor TGA	9563	5811	0.7
Solyc05g009660.1.1	K14431	Transcription factor TGA	1746	1997	−0.2
Solyc10g080410.1.1	K14431	Transcription factor TGA	85	1312	−4.0
Solyc10g080780.1.1	K14431	Transcription factor TGA	4146	6741	−0.7
Solyc04g072460.1.1	K14431	Transcription factor TGA	7045	14,224	−1.0

#### Auxin

The results presented in Table 5 show that 19 auxin-related genes had a higher expression level in the FAZ (Group B), and 20 auxin-related genes had a higher expression level in the LAZ (Group A). This included auxin responsive genes, such as *Aux/IAA, Gretchen Hagen3* (*GH3*), and *Small Auxin Upregulated RNA* (*SAUR*), auxin response factor (*ARFs*) genes, and auxin transport-related genes, such as *Like Auxin* (*LAX*) influx carriers and *Pin-formed* (*PIN*) efflux carrier genes. Aux/IAA proteins are negative repressors which bind to the Auxin-Responsive Elements (AREs) of the target gene promoters, leading to activation or repression of the target genes, and their degradation is promoted by auxin (Worley et al., 2000; Overvoorde et al., 2005). The members of the tomato *Aux/IAA* gene family were differentially regulated in the tomato FAZ and LAZ (Table 5). The *SlIAA6,9,25,26,14* genes were overexpressed in the FAZ, with *SlIAA14* showing the highest expression among this family members. On the other hand, *SlIAA8,10,23,24,21,3* were overexpressed in the LAZ, with *SlIAA24* showing the highest expression level among this family members. The *Aux/IAA1,3,4,7,8,9*,10 genes were downregulated in tomato FAZ and served as good markers for auxin depletion after flower removal (Meir et al., 2010). Similarly, *CitAux/IAA3,4,18,19* genes were downregulated in citrus fruit AZ during fruitlet abscission (Xie et al., 2015). Genetic mutation and expression analysis demonstrated that *ARF* genes could regulate plant organ abscission (Ellis et al., 2005; Guan et al., 2014). The results presented in Table 5 show that *SlARF18,1,3,9B* genes were over-expressed in the LAZ, whereas *SlARF5,19* genes were over-expressed in the FAZ. The expression of *SlARF1* was the highest in both AZs compared to the other family members of this gene.

The tomato homolog of *METHYLESTERASE1* (*MES1*) (AK328818, Solyc03g070380) was highly expressed in the LAZ (Group B) compared to the FAZ (Group A), whereas the *DWARF IN LIGHT1* (*DFL1*)/*auxin-inducible GH3.9* gene (AK319847, Solyc07g063850) was expressed equally at very high levels in both AZs (Group C) (Table S3). *MES1* converts the storage form of IAA to the active free form, whereas *DWARF IN LIGHT1* does the opposite, i.e., it converts the active free IAA to the inactive conjugated form (Staswick et al., 2005; Woodward and Bartel, 2005; Yang et al., 2008; Ludwig-Müller, 2011). In addition, conjugation of IAA to amino acids provides a negative feedback loop for controlling auxin homoeostasis, and the *SlGH3-2,10,15* genes, which control IAA conjugation, were found to respond quickly to exogenous auxin application (Kumar et al., 2012; Meir et al., 2015). Our KEGG analysis revealed that *SlGH3-2,10* genes were overexpressed in the FAZ, whereas *SlGH3-15* was over-expressed in the LAZ (Table 5).

The auxin influx and efflux transporters PIN and AUX/LAX proteins mediate the auxin polar transport (Vanneste and Friml, 2009), resulting in directional auxin flow and creation of auxin gradients (Bainbridge et al., 2008; Petrásek and Friml, 2009). The auxin influx carrier genes, *SlLAX3,4*, were overexpressed in the tomato FAZ compared to the LAZ, whereas *SlLAX5* was expressed at low levels in both AZs, but was comparatively expressed higher in the LAZ (Table 5). The auxin efflux carrier genes, *SlPIN1,4,9* were over-expressed in the FAZ compared to the LAZ, whereas *SlPIN3*,5,6,7 were over-expressed (by ~2- to 10-fold) in the LAZ compared to the FAZ (Table 5). Reduced auxin levels were attributed to increased activity of auxin efflux transporters in Arabidopsis and other systems (Sorefan et al., 2009; Meir et al., 2015).

#### Ethylene

Many genes related to different steps of the ethylene signaling transduction pathway were expressed in both the tomato AZs following flower removal and leaf deblading (Table 5). Out of six genes encoding for ethylene receptors in tomato (Klee, 2002, 2004), four genes were presented in both AZs. Among them, *ETR3,4,5* were expressed at very high levels in the LAZ, whereas *ETR1* was expressed higher in the FAZ (Table 5). These data confirm previous results demonstrating the expression of *ETR1,4* genes in tomato FAZ during pedicel abscission (Payton et al., 1996; Meir et al., 2010). The *Constitutive Triple Response1 (CTR1)* gene product acts downstream of the ethylene receptors, and belongs to the Arabidopsis *RAF Mitogen-Activated Protein Kinase Kinase* (*MAPKKK*) subfamily, which negatively regulates ethylene signal transduction (Kieber et al., 1993). Recent studies of genome-wide analysis of the *Mitogen-Activated Protein Kinase* (*MAPKK*) and *MAPKKK* gene families in tomato revealed five and 89 genes, respectively (Wu et al., 2014). The predicted transmitter domain of *ETR1* and the regulatory domain of *CTR1* were found to interact directly (Clark et al., 1998; Huang et al., 2003; Binder, 2008). Our analysis data show that three *CTR* genes, *CTR1*-*SlMAPKKK77, CTR3*-*SlMAPKKK68*, and *CTR4*- *SlMAPKKK78*, were expressed in both AZs, with *CTR1* showing the highest expression (Table 5). All the *CTR* genes identified in this study belong to the *RAF MAPKKK* sub-family according to the classification detailed previously (Wu et al., 2014).

Downstream ethylene signaling events are mediated by ERFs, which are plant-specific TFs which belong to the large AP2/ERF super-family (Riechmann et al., 2000), containing a *cis*-acting ethylene-responsive element named the GCC-box in their promoter regions (Fujimoto et al., 2000). The GCC-box interacts with *trans*-acting factors termed ethylene-responsive element-binding proteins, which are required for ethylene regulation in many plant species (Ohme-Takagi and Shinshi, 1995). Our data show that *SlERF.C1* and *SlERF.E2* genes were highly expressed in the FAZ, while *SlERF.C2* and *SlERF* (Solyc11g011740) genes were highly expressed in the LAZ (Table 5). Interestingly, two other *ERF* genes (Solyc05g051180, Solyc11g011750) were present only in the LAZ (Group A1) (Table 5). The *ERF1c* gene (AY044236, *SlERF.C1*) was proposed to be involved in the late stages of flower pedicel abscission (Meir et al., 2010). Several other tomato *ERF* genes, *SlERF52* (Solyc03g117130), *SlERF56* (Solyc09g066360), *SlERF68* (Solyc08g078180), *ERF1* (AF502085.1, *SlERF.E2*), and *ERF2* (AI776626, Solyc09g089910), were overexpressed in both the FAZ and the LAZ (Table S3).

*Ethylene Insensitive2* (*EIN2*) was reported to act downstream of *CTR1* as a positive regulator of the ethylene signaling pathway (Alonso et al., 1999). *LeEIN2* was expressed in both the FAZ and the LAZ at similar levels (Table 5). *EIN3*, which acts downstream of *EIN2*, belongs to a multi-gene family designated as *Ethylene Insensitive-Like* (*EIL*) in tomato. *EIN3* encodes a downstream component of the ethylene signal transduction pathway, which ultimately activates ethylene-responsive genes (Ecker, 1995; Roman et al., 1995). *LeEIL*s are functionally redundant and positive regulators of multiple ethylene responses throughout plant development. (Tieman et al., 2001). Our data show that all the *EIL* genes, *LeEIL1,2,3,4*, were expressed at high levels in both AZs, with the highest levels in the FAZ (Table 5).

#### Other hormones

Several genes involved in pathways related to other phytohormones were expressed in both the FAZ and the LAZ (Table 5). Thus, a gene involved in JA perception and signaling *COI1* was highly expressed in the FAZ (Table 5). This gene encodes an F-box protein, which is required for JA signaling in Arabidopsis (Xie et al., 1998). A gene involved in brassinosteroid biosynthesis and signaling encoding for the BRI1 protein—*tBRI1/SR160* (Koka et al., 2000; Montoya et al., 2002), showed a high expression in both the FAZ and the LAZ, with the highest level in the LAZ (Table 5). A gene involved in GA signaling, *LeGAI* (Martí et al., 2007; Bassel et al., 2008), also showed a higher expression in the LAZ compared to the FAZ (Table 5).

### Designing and preparation of an AZ-specific microarray for transcriptomic abscission research in tomato

The above comparisons between gene expression in the FAZ and the LAZ were based on samples pooled from individual samples taken at the different time points during pedicel and petiole abscission following abscission induction (Figure 1). The pooled samples limit the identification of specific gene expression profiles at the timing of the spatial events of the abscission process. To perform an analysis that enables to reveal the sequences of the molecular events involved in petiole and pedicel abscission following induction by artificial auxin depletion (IAA source removal), it is necessary to develop a low-cost method than the RNA-Seq, but with comparable robustness, to perform transcriptomic analyses. For this purpose, we developed an AZ-specific microarray based on the RNA-Seq results.

The RNA-Seq analysis of the tomato FAZ and LAZ samples revealed a total number of 40,959 transcripts, including 31,298 transcripts analyzed for sequence similarity to the known database of sequences, 8823 novel tomato transcripts (novel ORF predictions), and 838 transcripts annotated with *A. thaliana* and *N. tabacum* (Table 6). The NGS annotated and the novel transcripts from the tomato FAZ and LAZ, together with sequences of known abscission-related genes originated from various sources detailed in Table 6, were used to design the AZ-specific microarray chip. We included all these available transcripts to enrich this customized microarray chip with previously reported AZ-related genes. As a result, the eArray was finalized with 100,276 probes for the 41,315 transcripts (Table 6).

**Table 6:** **Probe design summary for the AZ-specific microarray chip**.

**Transcript category**	**No. of transcripts**	**No. of probes per transcript**	**Total No. of probes**
Annotated transcripts (RNA-Seq)	31,298	2 (1 sense and 1 antisense)	62,596
Novel transcripts (RNA-Seq)	8823	4 (2 sense and 2 antisense)	35,292
Annotated with *Arabidopsis thaliana & Nicotiana tabacum*	838	2 (1 sense and 1 antisense)	1676
Total No of transcripts resulted from RNA-Seq study	40,959		
Previously reported transcripts related to the abscission process	356	2 (1 sense and 1 antisense)	712
Total No. of transcripts for Tomato Array	41,315		100,276
No. of probes collected after redundant/ probe selection criteria			93,674
Additional transcripts for technical quality control	50	4 (2 sense and 2 antisense)	200
No. of Agilent probes added in the Array		2 (1 sense and 1 antisense)	2278
Total No. of probes designed for Tomato Array	(93,674+200 + 2278) = 96,152		
Total no of probes replicated to fill the remaining spots#	79,874		
Total no of probes in the final array	176,026		

*The transcripts were obtained from various sources: RNA-Seq annotated and novel transcripts of the FAZ and LAZ prepared as detailed in Tables 1, 2; previously reported transcripts related to abscission (Meir et al., 2010; Wu et al., 2011), and additional transcripts obtained from Mark L. Tucker (USDA-ARS, USA). The probe was designed specifically for both sense and antisense orientations for the specific transcripts. The microarrays were designed for the transcripts listed in the table, using the Agilent custom gene expression microarray, 4X180K. The microarray was finalized with 176,026 probes including the technical quality and Agilent control grid probes*.

A 4x180K gene expression array was designed with the probes having 60-mer oligonucleotides from annotated and novel transcriptome sequenced data and gene sequence related to *Nicotiana tabcum* and *A. thaliana*. The 4x180K array comprised of 180,880 features including 176,026 probes and 4854 Agilent controls. All the oligonucleotides were designed and synthesized *in situ* according to the standard algorithms and methodologies used by Agilent Technologies for 60 mer *in situ* oligonucleotide DNA microarray (Table 6). The probes were designed in both sense and antisense orientations and with multiple probes for each transcripts (Table 6, Figure S5). Blast analyses were performed against the complete set of sequence databases to check the specificity of the probes. Finally, 96,152 probes were designed, and 79,874 specific probes were duplicated to fill the remaining spots (Table 6). The detailed list of transcripts, probes, and cross hybrid probe details is presented in Table S11. The designed AZ-specific microarray chip (AMADID: 043310; Genotypic Technology Private Limited, India) is now available from the company for abscission research upon our approval.

### Validation of differentially regulated genes in the LAZ and the FAZ

**Figure 8:**
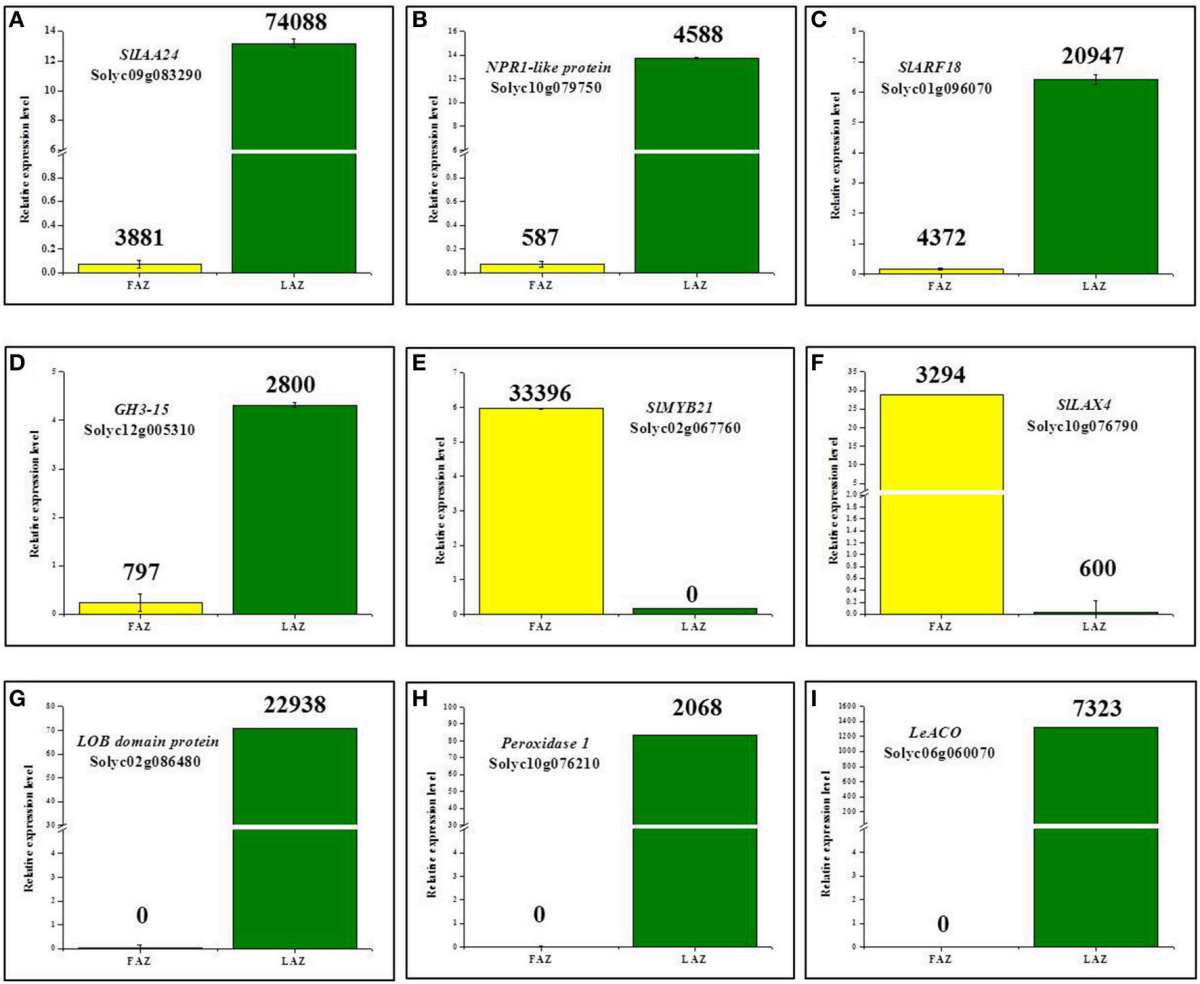
**Validation by qPCR of differential expression patterns of selected genes in the FAZ and LAZ pooled samples following abscission induction**. Expression levels were measured for tomato *Aux/IAA24* (*SlIAA24*) **(A)**, *NPR1-like protein* **(B)**, *Auxin response factor18* (*SlARF18*) **(C)**, *Gretchen Hagen3-15* (*GH3-15*) **(D)**, *SlMYB21* **(E)**, *Like Aux4* (*SlLAX4*) **(F)**, *LOB domain protein* **(G)**, *Peroxidase1* **(H)**, and *1-Aminocyclopropane-1-Carboxylate-Oxidase* (*LeACO*) **(I)**. The relative quantification of the gene expression level in the qPCR assay was determined by the comparative C_T_ method 2^−Δ*ΔC*^T, using *ACTIN* as a reference gene. The results are means of three biological replicates ± SD. The values presented on top of each bar indicate the expression levels derived from the RNA-Seq data. Transcript identities are indicated in the graphs by their gene ID. The qPCR and RNA-Seq analyses were performed with different samples taken from independent biological replicates of two separate experiments.

To verify the results of the RNA-Seq analyses, we characterized the expression patterns of nine arbitrarily selected genes, which were differentially regulated in the LAZ and the FAZ, using quantitative reverse transcription-PCR (qRT-PCR). We randomly selected the genes from the four differentially expressed groups, Group A, Group A1, Group B, and Group B1 (Figure 2). In the tomato AZs, the expression levels of the auxin-related genes, *SlIAA24, SlARF18, GH3-15*, and *NPR1-like protein* (Solyc10g079750) mRNAs, highly increased in the LAZ (Group A) (Figures 8A–D). In Group A1, the *LOB domain protein*s (Solyc02g086480), *Peroxidase1* (Solyc10g076210), *1-aminocyclopropane-1-carboxylate oxidase* (Solyc06g060070), were exclusively expressed in the LAZ (Figures 8G–I). The auxin influx carrier gene *SlLAX4* (Solyc10g076790) was highly expressed in the FAZ (Group B) (Figure 8F). In the group of genes exclusively expressed in the FAZ (Group B1), a MYB TF, *SlMYB21* (Solyc02g067760) was highly expressed, but it also exhibited a low expression in the LAZ (Figure 8E), which was not spotted by the RNA-Seq analysis. The qPCR performed in this study for the selected genes confirms of the RNA-Seq data. The slight variations obtained between the qRT-PCR and RNA-Seq results are due to the newly pooled samples used for the qRT-PCR, similar to the method used for the RNA-Seq analysis. It seems, therefore, that in general the qRT-PCR technique validated the RNA-Seq data of expression patterns of all the selected transcripts in pooled samples of the LAZ and the FAZ during the abscission process.

## Discussion

The present study was based on examination of the effects of auxin depletion, obtained by organ removal, on the molecular changes occurring during the abscission process in tomato FAZ and LAZ (Figure 1). Quantitative measurements of the changes in endogenous auxin content following flower removal were already performed in tomato FAZ, and indeed showed a significant reduction in auxin content with time (Guan et al., 2014). Basically, our study included two parts: (1) Analysis of the RNA-Seq transcriptome with the pooled samples strategy and the preparation of the customized AZ-specific microarray based on it; (2) Use of the transcriptome data for comparative analysis of global gene expression in the tomato FAZ and LAZ. The customized AZ-specific chip was further used for specific analyses at each time point with the appropriate replicates. Part of the microarray results was recently published elsewhere (Kim et al., 2015; Meir et al., 2015), and part are still being analyzed. Hence, the present study does not include the microarray data, as its main aim was to expose the occurrence of global changes.

### Transcriptome assembly and design of an AZ-specific array

Transcriptome studies were previously performed in the tomato FAZ and flower NAZ using microarrays (Meir et al., 2010, 2011; Nakano et al., 2012, 2013; Wang et al., 2013; Ma et al., 2015). However, a comparative transcriptome profiling of the FAZ and the LAZ was not performed, and no comprehensive cell separation stages in the FAZ (0–14 h after flower removal) and the LAZ (0–96 h after leaf deblading) were addressed by RNA–Seq. The aim of the present research was to study the transcriptome of tomato FAZ and LAZ pooled samples of the various time points, using Illumina PE sequencing and *de novo* assembly to permit assessment of expression across the entire genome. In addition, the data generated were used to create an AZ-specific microarray chip that enables to study the specific abscission stages. We have utilized both the *de novo* as well as the reference-based assemblies of tomato transcriptome to generate a more comprehensive and unbiased representation of the FAZ and the LAZ pooled samples during tomato flower and leaf abscission process. Stringent categories were applied for both the generated *de novo* assembly (BLAST with 50% identity and 70% query coverage) (Tables S5, S6) and the reference-based assembly (BLASTN with *E*-value of e^−5^). It should be noted, that after the genome alignment with the current tomato reference genome, the analysis revealed only 76 and 90 unannotated transcripts (FASTA file) for the FAZ and the LAZ, respectively. These results suggest that the sequences generated are of tomato genes rather than sequences of non-coding RNAs, UTRs, unannotated introns, or contaminated sequences from other organisms, such as fungi, bacteria, and viruses. The assembly statistics revealed that the merged assembly had better characteristics, such as higher N50 value, average, and longest contig length, total number of contigs, and etc., which usually serve as hallmarks to assess the assembly quality (Table S2). The coverage of the transcriptome was comprehensive enough to discover major genes of several metabolic and regulatory pathways. Particularly, genes associated with plant hormonal signaling components could be mapped to their relevant pathways.

We used the strategy of transcriptome sequencing (RNA-Seq) of pooled samples (RNA pooled equally from all time points), since there are specific transcripts of various transcriptional networks and genes of various functions which are induced at specific time points in the AZ (Meir et al., 2010). The pooling strategy ensures that a given transcript will be present in abundance during the RNA-Seq analysis, rather than analyzing it at a specific time point in which it might not be highly expressed. In the present study, we compared the global transcriptomes generated from the FAZ and the LAZ rather than performing a direct quantitation of the expressed genes in each abscission stage. The differential expression levels described in this study are related to the fold changes of the transcripts in the FAZ and the LAZ pooled samples. However, by pooling samples from different time points during the abscission process we lost the resolution to detect dynamic and specific changes in gene expression at specific time points. Hence, for the differential expression analysis, we utilized the transcriptome data to generate an AZ-specific microarray chip, which allowed a cost effective way to detect all the dynamic changes in gene expression at all stages of the abscission process. We had to design this AZ-specific chip, since the commercial Affymetrix Tomato Gene Chip used in our previous studies (Meir et al., 2010) contained only about 10,000 genes, and many AZ-specific genes were not included in this array. Transcriptome sequencing data were previously utilized to create customized microarrays suitable for various research needs in Syrian golden hamsters (Ying et al., 2015) and *Camellia sinensis* (Wang et al., 2014a). Customized microarrays are used for global transcriptome analysis in various organisms/systems (Lipovich et al., 2014; Yasuike et al., 2015), and strand-specific customized arrays were designed for studying NATs in the Human genome (Yelin et al., 2003) and mouse (Kiyosawa et al., 2005).

The additional novelty of our AZ-specific microarray is that the probes were designed in both sense and antisense orientations, and multiple probes were used for each transcripts (Table 6, Figure S5). Thus, this developed tomato AZ-specific chip contains much more transcripts than any other commercial microarray chip. Therefore, it can serve as an excellent means to further study the tomato AZ transcriptome. We have utilized the AZ-specific microarray chip (GSE45355; GSE45356; GSE64221) for the transcriptome analysis of several genes in the tomato FAZ and LAZ samples. Part of these analyses at specific time points following abscission induction, which represent various abscission stages, was already published. These publications showed changes in expression of specific groups of tomato genes, such as auxin-related genes in the FAZ and LAZ (Meir et al., 2015), and genes associated with cell wall, boundary layer, pathogen-related, and lipid transport in the FAZ and NAZ (Kim et al., 2015; Meir et al., 2015). Regarding the clear advantages of this AZ-specific microarray, these recent studies enabled the exploration of genes responsible for abscission induction, execution and synthesis of the defense layer processes in the tomato LAZ and FAZ.

The rapid technological progress in NGS, which led to the generation of the Strand-specific RNA-Seq, can provide a bp resolution. Thus, several cheap library construction protocols are already available (Zhong et al., 2011), and can be utilized for similar research needs. Our choice of a customized microarray platform was driven by cost advantages relative to RNA-Seq, for robustness of its own kind with well-standardized methods, and by the known inadequacies of commercial microarray platforms, which under-represents genomic complexity.

### Annotation analysis characterizes abscission as a dynamic process

The GO terms of “protein binding,” “oxidation-reduction process,” and “membrane” were the most represented ones among the categories of molecular function, biological process, and cellular component, respectively, in both tomato AZs (Figure 3). The data show that both the FAZ and the LAZ share a similar type of gene enrichments in all the three categories of biological process, molecular function, and cellular component, with only few differences, when samples of all-time points following abscission induction were pooled together. These annotations of the over-expressed categories provide a useful basis for studying gene functions, cellular structures, and processes in the two examined AZs (Tables S8, S9). Our data are consistent with the data of the Arabidopsis stamen AZ (Cai and Lashbrook, 2008) and olive fruit AZ transcriptomes (Gil-Amado and Gomez-Jimenez, 2013) in the category of cellular component, where the GO term “membrane” is the most represented. In the olive fruit AZ transcriptome, the most represented term in the category of biological process was metabolic process; whereas in the tomato FAZ and LAZ our data show that the terms of “oxidation-reduction process” and “metabolic process” were represented at similar levels (Figure 3). In the laminar AZ of citrus leaves, the most important GO terms represented cell organization, biogenesis/metabolic process, metabolism of fatty acids, carbohydrate metabolism, response to biotic and abiotic stimuli, and transport (Agustí et al., 2012). Our data in tomato (Tables S8, S9, Figure 4) confirm that these terms were highlighted in the over-expressed groups in the LAZ (Group A). This is in agreement with earlier studies of the abscission process in Arabidopsis and olive (Cai and Lashbrook, 2008; Gil-Amado and Gomez-Jimenez, 2013), suggesting that most genes involved in AZ functions are being shared at all stages of the abscission process of various organs, although there might be significant changes in the transcriptional activities at each stage of the abscission process.

### Comparative gene expression in the tomato FAZ and LAZ

#### Transcription factors gene families

TFs play key roles in plant development and act as major switches of various transcriptional regulatory networks by temporarily and spatially regulating the transcription of their target genes. Recent reports highlighted the involvement of various TFs in organ abscission and dehiscence processes, FAZ development, and formation of protective layers (Meir et al., 2010, 2011; Nakano et al., 2012; Corbacho et al., 2013; Gil-Amado and Gomez-Jimenez, 2013; Parra et al., 2013; Liu et al., 2014). Since many genes are involved in the abscission process, it is not feasible to manipulate such a complex and dynamic process by modifying a single gene expression, and therefore efforts have been focused on specific TFs that control the entire pathways (Nath et al., 2007; Van Nocker, 2009; Ma et al., 2015). Many TFs families associated with FAZ development during tomato flower abscission process was already characterized, and it was interesting to study if these TFs families are also involved in tomato leaf abscission.

MYB is one of the largest plant TF families (Riechmann et al., 2000), which is involved in various regulatory pathways (Jin and Martin, 1999). Our results show that several MYB genes were upregulated and exclusively present in the FAZ and LAZ (Table S10). In tomato, it was shown that the Blind (*Bl*) gene, which encodes the R2R3 class MYB TF, was upregulated in the AZ-forming lines (Nakano et al., 2012), and it interacted with *jointless* in controlling the meristem cell fate (Schmitz et al., 2002; Quinet et al., 2011). In rice, a single non-synonymous substitution (G to T) in the shattering gene *sh4*, encoding a MYB3 DNA-binding domain, resulted in AZ disfunction and incomplete development, leading to reduced seed shattering (Li et al., 2006). Most members of the MYB families were shown to be abundantly present in melon fruit AZ (Corbacho et al., 2013) and were upregulated in olive fruit AZ (Gil-Amado and Gomez-Jimenez, 2013) during fruit abscission. Additionally, our data show that *SlMYB43*—*THM16* (Solyc11g011050), which is a homolog to *AtMYB43* and *PtrMYB152*, was present in both tomato AZs and overexpressed in group A (Table S10). This indicates that this gene regulates secondary cell wall biosynthesis similarly to Arabidopsis MYBs (Wang et al., 2014b). Other MYB genes, such as *SlMYB3* (Solyc06g065100)—*At1g22640* (salicylic acid- and ABA-inducible TF), *SlMYB108* (Solyc12g099130)—*AT3G06490* (ethylene- and JA-inducible TF), were expressed in both tomato AZs (Table S10). This supports the idea that these MYB proteins act as critical components of multiple hormone-mediated transcriptional cascades and cell wall biogenesis, which regulate tomato flower and leaf abscission.

ZF proteins regulate many developmental and stress responses (Takatsuji, 1998, 1999). Our results show that 98 ZF TF genes were overexpressed in both AZs, being the second most highly expressed family in Groups A and B (Figure 7A, Table S10). In Arabidopsis, *ZINC FINGER PROTEIN2* was upregulated in the stamen AZ, and its overexpression delayed the abscission process and contributed to the AZ development (Cai and Lashbrook, 2008). The C2C2 type ZF genes were upregulated in the tomato FAZ upon ethylene-induced abscission (Wang et al., 2013).

Most of the MADS-box and GRAS TFs gene families were expressed in both tomato AZ tissues represented by Groups A and B (Figure 7A, Table S10). Since our pooled LAZ and FAZ samples were taken when the AZs were already well defined, the higher expression of MADS-box and GRAS TFs gene families in these samples suggest that these TF genes also regulate the late stages of the abscission process, when the AZ is already differentiated. It was previously proposed that MADS box TFs might substantially contribute to the specificity of the identity of the pedicel regions (Nakano et al., 2012), and might form region-specific protein complexes similar to the floral quartets model of flower organ identification (Theissen and Saedler, 2001). Our data show that the MADS-box gene *MACROCALYX* (Solyc05g012020) is highly expressed in the tomato FAZ (Group B), whereas the *JOINTLESS* (Solyc11g010570) gene was expressed at a similar level in both the FAZ and the LAZ (Group C) (Table S10). Therefore, we speculate that a similar type of organ identity specification, reported for the FAZ, might also operate in the LAZ.

Our results show that 137 *bHLH* TFs genes, out of 159 identified *bHLH* genes in tomato, were overexpressed in both the FAZ and the LAZ (Table S10). These genes were distributed among all the groups presented in Figure 2, indicating that the bHLH TF family is associated with the abscission process. It should be emphasized that our expression studies were performed with pooled samples, and therefore they do not represent the pattern of abscission progress. Most members of the *bHLH* TF gene family were downregulated during abscission of mature olive fruit (Gil-Amado and Gomez-Jimenez, 2013). Similarly, the *bHLH* TF gene (AW648468) was sharply downregulated in the tomato FAZ after flower removal (Meir et al., 2010). Mutation in the *myc/bHLH* gene *ALCATRAZ* in Arabidopsis delayed fruit dehiscence by blocking the separation of the valve cells from the replum (Rajani and Sundaresan, 2001). MYB and bHLH proteins (MYC type bHLH) interact to form multi protein complexes to regulate gene transcription (Wolberger, 1999; Zimmermann et al., 2004). It seems therefore, that *bHLH* and *MYB* TFs manifest a potential interaction necessary for the regulation of genes operating in downstream events in the FAZ and the LAZ during flower and leaf abscission.

The majority of the bZIP TFs were present and overexpressed in the tomato LAZ (Group A, A1) (Figure 7, Table S10). Among the bZIP TFs, the *HY5* (Solyc08g061130) gene was present and equally expressed in both AZs (Group C). Most members of the bZIP family genes and *HY5* were specifically induced and abundantly present in melon fruit AZ (Corbacho et al., 2013), and were upregulated in olive fruit AZ during abscission (Gil-Amado and Gomez-Jimenez, 2013; Parra et al., 2013). The *bZIP* gene (BG631669) was downregulated at an early stage of tomato pedicel abscission (Meir et al., 2010). The TGA type *bZIP* genes were found to be involved in plant development (Izawa et al., 1993), auxin-induced stress responses (Pascuzzi et al., 1998), and regulation of abscission-specific *Cel* gene expression (Tucker et al., 2002). This suggests that these TF genes might act as positive regulators of abscission signaling. Our results suggest that different bZIP TFs probably mediate the abscission-responsive transcription processes in flowers and mainly in leaves. Taken together, our data corroborate that in the tomato FAZ and LAZ, TFs belonging to these families may potentially act to trigger the transcriptional cascade during abscission and formation of the defense layer. Additional research is needed to reveal the molecular basis of the regulation of expression of these genes.

#### Key meristem genes

Reports demonstrating the expression of key meristem genes and their functional association with the AZ are emerging, implying that the undifferentiated AZ cells have the capability to differentiate in response to various stimulators. Most of the key meristem genes were expressed in both tomato AZs (Table 3). The *Tkn3*/*KNAT6, TBL4, LBD1, Bl* were highly expressed in the tomato LAZ (Group A) compared to the FAZ (Table 3). The KNOX family genes regulate the size and proliferation of the AZ cells during floral organ abscission (Shi et al., 2011). Moreover, *TKN3* and *BL4*, encoding KNOX and BELL family TFs, form a heterodimer required for SAM functioning (Rutjens et al., 2009). On the other hand, the *WUS, OFP, MYB-Cpm10/MYB78, Goblet, Ls* were highly expressed in the tomato FAZ compared to the LAZ (Group B) (Table 3). *WUSCHEL-related homeobox-containing protein* (*WUS*) and *KNOX* gene families are key genes in the regulation of the maintenance of undifferentiated cells (Long et al., 1996; Mayer et al., 1998; Lenhard et al., 2002). It was previously reported that the homologs of *WUS* and its potential functional partner *OFP* were downregulated during flower abscission (Meir et al., 2010; Wang et al., 2013). More interestingly, the family members of Arabidopsis *KNAT6, BELL-like homeodomain protein 1*, and *OFP*, can potentially form ternary complexes, which are critical for meristem activities (Hake et al., 2004; Hackbusch et al., 2005; Hamant and Pautot, 2010; Li et al., 2011). Similarly, homologs of *LBD1* (Wang et al., 2013) and the SAM gene, NAC-domain TF *GLOBLET* (Berger et al., 2009), were also highly expressed in the tomato FAZ (Hu et al., 2014). *WUS, Bl*, and *Ls* genes displayed differential expressions between tomato wild type and the *mc* mutant, and hence showed a *jointless* phenotype (Nakano et al., 2012). On the other hand, *Bl* and *AGL12* were continuously and specifically induced in the tomato FAZ during the pedicel abscission process (Meir et al., 2010; Wang et al., 2013). The *REVOLUTA* gene is involved in apical meristem development for limiting cell divisions in Arabidopsis (Talbert et al., 1995). Overexpression of a microRNA166-resistant version of the tomato *REVOLUTA* (*SlREV*) (*35S::REV*^*Ris*^) caused dramatic reproductive alterations, including continuous production of flowers at the FAZ (Hu et al., 2014). Our data show that *SlREV* (Solyc11g069470) was expressed equally (Group C) in both the FAZ and the LAZ samples (Table S3), indicating its importance for the development of the apical meristem in both AZs.

Our findings showing that most of the SAM and auxiliary meristem genes are preferentially expressed at differential levels in both the tomato FAZ and LAZ and interact with each other, support the idea that meristem activity genes play important roles in maintaining the undifferentiated status of cells in both AZs. The transcriptome data show that there are substantial differences between the tomato FAZ and LAZ. In particular, genes involved in key meristem functions show distinct expression patterns. These differences between the FAZ and the LAZ might explain the significant differences observed in the rate of pedicel and petiole abscission, respectively, when petiole abscission took a much longer time and had to be enhanced by ethylene (Figure 1). Apart of the key meristem genes, many other floral meristem genes were preferentially expressed in both the tomato FAZ and LAZ (Table S3).

#### Cell wall related genes

Our transcriptome analyses showed that the majority of cell wall degrading and remodeling factors, including *PG, Cel, XTH*, and *EXP* genes (Table 4) were highly expressed in both tomato AZs. These genes were previously demonstrated to be highly expressed also in the AZs of a large number of abscission systems including tomato AZ (Lashbrook et al., 1994; del Campillo and Bennett, 1996; Cho and Cosgrove, 2000; Taylor and Whitelaw, 2001; Roberts et al., 2002; Agustí et al., 2008; Ogawa et al., 2009; Meir et al., 2010). *TAPG1* was highly expressed in the LAZ following exposure of the debladed leaf explants to ethylene (Table 4), thereby confirming previous reports showing that *TAPG1* transcript in the LAZ was induced by ethylene (Jiang et al., 2008) and inhibited by auxin (Hong et al., 2000). In addition, *TAPG4* showed the highest expression level in the FAZ, and therefore its promoter was used for specific silencing of genes in the tomato FAZ (Ma et al., 2015). Previous microarray experiments performed with the tomato pedicel abscission system showed that *Cel1* and *Cel5* were strongly and specifically upregulated in the FAZ, but the expression levels of *Cel2,3,7,8* were very low in the FAZ and were not affected by flower removal (Meir et al., 2010). However, our results demonstrate that the *Cel2,5,6,7,8* genes were present and expressed at high levels in the LAZ pooled samples (Table 4). The functions of *Cel1* and *Cel2* were already demonstrated by antisense suppression in tomato flower and leaf abscission (Lashbrook et al., 1998; Brummell et al., 1999). *Cel1,6,9* genes were upregulated in the LAZ of soybean (*Glycine max*) explants after ethylene treatment (Tucker et al., 2007). Expansins are involved in cell wall enlargement and pectin remodifications (Lee et al., 2001; Cosgrove et al., 2002; Zenoni et al., 2011), and were reported to regulate pedicel abscission in Arabidopsis and soybean and leaflet abscission in elderberry (*Sambucus nigra*) (Cho and Cosgrove, 2000; Belfield et al., 2005; Tucker et al., 2007). We observed that the expression levels of several *EXP* genes, *LeEXP1,5,9,11,18*, were particularly high in the LAZ (Table 4). It was previously reported that the expression levels of *EXP3*,4,5,9 genes in tomato, which were specifically and highly expressed in the FAZ, decreased during the process of pedicel abscission, and were not affected by the 1-MCP pretreatment (Meir et al., 2010). *LeEXP1,11* genes were also specifically upregulated in tomato FAZ (Wang et al., 2013).

Taken together, our data regarding the changes in the expression of genes encoding cell wall modifying enzymes, confirmed previous reports on various abscission systems. The results also indicate that both the FAZ and the LAZ have similar types of cell wall-related genes, but with different expression levels, which were generally higher in the LAZ than in the FAZ. This may be ascribed to the ethylene treatment applied to the debladed leaf explants in order to enhance the rate of leaf petiole abscission (Figure 1B). Ethylene treatment might also induce an increased expression of cell wall modifying genes in the LAZ.

#### Hormonal signal transduction genes

The interplay of plant hormones during the abscission process has been widely reported. In tomato, the pedicel abscission process is inhibited by a continuous auxin flow from the flowers, and is triggered by ethylene following auxin depletion in the FAZ (Roberts et al., 1984; Meir et al., 2010). Ethylene also inhibits auxin transport, thereby enhancing auxin depletion in the AZ (Meir et al., 2015). Therefore, it was expected that genes of auxin and ethylene signal transduction cascades would be expressed predominantly in both the FAZ and the LAZ as compared to the other plant hormones. A continuous auxin flow to the AZ is required for preventing the acquisition of ethylene sensitivity by the AZ cells, which leads in turn to organ abscission (Taylor and Whitelaw, 2001). The molecular basis of auxin transport in tomato was elucidated in sympodial growth, compound leaves, fleshy fruit, and whole plants (Giovannoni, 2004; Kimura and Sinha, 2008; Nishio et al., 2010; Pattison and Catalá, 2012). This auxin flow is presumably regulated by auxin-responsive genes, belonging to three major groups, *Aux/IAA, SAUR*, and *GH3*. These auxin-responsive genes, which are regulated by ARFs (Guilfoyle and Hagen, 2007), are well characterized in tomato (Kumar et al., 2011, 2012; Wu et al., 2012). Our data show that various auxin-related genes, such as *Aux/IAA, SAUR, GH3, ARFs, LAX*, and *PINs*, are expressed in both the tomato FAZ and LAZ at various levels (Table 5).

The expression level of *Aux/IAA* genes was used as a marker for auxin activity associated with inhibition of floret abscission by auxin application in *C. elegans* (Abebie et al., 2008), as well as a marker of auxin depletion in tomato FAZ following flower removal (Meir et al., 2010) and in citrus AZ during fruitlet abscission (Xie et al., 2015). We previously showed that the auxin responsive genes *IAA1,3,4,7,8,9,10* were downregulated following abscission induction in tomato flowers (Meir et al., 2010), and our present data showed that *IAA3,8,9,10* were presented and expressed in both the tomato FAZ and LAZ pooled samples at various levels (Table 5). The remaining genes which were already shown to be present in the FAZ, *IAA1,4,7* (Solyc06g053840, Solyc04g076850, Solyc06g053830, respectively), were shown to have equal expression in the FAZ and the LAZ (Table S3). These genes are not KEGG-annotated in the auxin hormonal pathway, which might indicate that that they are not significant for the auxin signaling cascade.

ARFs are TFs that bind to AREs in promoters of early auxin responsive genes and play central roles in many auxin-mediated processes, leading to activation or suppression of the selected genes. The expression pattern and the possible role of the *ARF* gene family in the tomato FAZ, as well as auxin- and ethylene-induced changes during flower abscission were comprehensively studied (Guan et al., 2014). We showed that multiple *ARF* genes were expressed in both tomato AZs (Table 5, Table S3). Upregulation of the *SlARF1,3,5,19* delayed the abscission process in the FAZ (Guan et al., 2014), indicating that these ARFs have a similar function in both AZs.

Several reports suggested that *GH3* genes are involved in flower or fruitlet abscission (Kuang et al., 2012; Wang et al., 2013; Meir et al., 2015). *MES1* converts the storage form of IAA into its active free form, whereas *Dwarf in Light1* does the opposite, i.e., converts active free IAA to an inactive conjugated form (Staswick et al., 2005; Woodward and Bartel, 2005; Yang et al., 2008; Ludwig-Müller, 2011). Our results demonstrate that *MES1* and *DFL1* (Table S3) as well as other *GH3* genes (Table 5) were expressed in both tomato AZs. Recently, we reported (Meir et al., 2015) that *GH3* genes in the FAZ were upregulated in response to auxin depletion, confirming that increased IAA conjugation is involved in the process of auxin depletion, while their expression decreased in the LAZ after leaf deblading, further confirming that *GH3* is an auxin-induced gene. In addition, conjugation of IAA to amino acids provides a negative feedback loop to control auxin homoeostasis, and the *SlGH3-2,10,15* genes, which control IAA conjugation, were found to respond quickly to exogenous auxin application (Kumar et al., 2012; Meir et al., 2015).

Reduced auxin levels in Arabidopsis were attributed to increased activity of auxin efflux transporters (Sorefan et al., 2009). Recently, it was demonstrated that the *KD1* gene played a role in modulating auxin levels in the tomato FAZ, by altering the expression profiles of the auxin efflux transporters, *PIN9* (HQ127075; *SlPIN9*) and *PIN-like3* (SL_TC197872) (Ma et al., 2015). Our data show that the auxin influx carrier genes, *SlLAX3,4*,5 and the auxin efflux carrier genes *SlPIN1*,3,*4,5,6,7,9*, were present in both the tomato FAZ and LAZ (Table 5). In addition, *SlPIN3*,5,6,7 genes were overexpressed (by ~2- to 10-fold) in the LAZ compared to the FAZ (Table 5). Most of the auxin-related gene families are expressed in both AZs, but some members of different gene families are expressed specifically in the FAZ or the LAZ (Meir et al., 2015). The only study so far on the effect of leaf deblading on expression of auxin-related genes in the LAZ was performed in *Mirabilis jalapa* (Meir et al., 2006). The differential expression of the auxin influx and efflux carrier genes between the two AZs suggests that different genes of these families play important roles in different AZs. This might further provide us with means for selective manipulation of leaf and flower abscission by specifically manipulating auxin transport in these two AZs. Such a manipulation might be important for agricultural purposes, for example to reduce olive fruit strength before harvest without affecting leaf abscission.

Ethylene response factors (ERFs) are plant transcriptional regulators that specifically bind the GCC motif of the promoter region of ethylene-regulated genes, thereby mediating ethylene-dependent gene expressions (Ohme-Takagi and Shinshi, 1995; Solano et al., 1998). These *SlERF* genes were reported to be specifically overexpressed in the tomato FAZ compared to the proximal (basal) and distal (apical) NAZ regions (Nakano et al., 2013; Wang et al., 2013). It was recently reported that in *SlERF52*-suppressed tomato plants, flower pedicel abscission was significantly delayed compared to the wild type, and accordingly, a reduced expression of the genes encoding for the abscission-associated enzymes, cellulase and PGs, was detected (Nakano et al., 2014). Our data show that several *SlERF* genes, and particularly *SlERF52*, which were reported to be essential for flower pedicel abscission, operate also in the tomato LAZ. The downstream components (positive) of ethylene signaling, such as *EIN2* and *EILS*, were expressed in both the tomato AZs (Table 5). Antisense lines of *LeEIL* plants exhibited delayed flower abscission (Tieman et al., 2001), which suggests that the *LeEILS* has the same function in the tomato LAZ.

Many genes related to different steps of the ethylene signaling transduction pathway were expressed in both the tomato AZs following flower removal and leaf deblading (Table 5). The tomato ethylene receptor genes *ETR1,3,4,5* were expressed in both AZs, confirming previous reports that they were expressed in the FAZ (Payton et al., 1996; Meir et al., 2010). The *Constitutive Triple Response1 (CTR1)* gene product acts downstream of the ethylene receptors, and negatively regulates ethylene signal transduction (Kieber et al., 1993). It was previously reported that *CTR1* was upregulated in early stages of the tomato pedicel abscission process, and was specifically expressed in the FAZ in the late stages of the abscission process (Meir et al., 2010). Our analysis data show that *CTR1,3,4* genes were expressed in both tomato AZs, with *CTR1* showing the highest expression (Table 5). This indicates that a similar cascade of events operates in both AZs.

## Conclusions

This study presents a *de novo* assembly of AZ-associated transcripts and provides valuable information about their functional annotation by using an integrated approach to enrich the transcriptome of *S. lycopersicum* FAZ and LAZ. To the best of our knowledge, this is the first comprehensive report on RNA-Seq of tomato FAZ and LAZ, which we believe would contribute to the understanding of the expression differences in these AZs. The present research identified genes that may be involved in the abscission process of tomato flowers and leaves, including genes encoding for TFs, hormone transduction components, cell wall-related enzymes, and key meristem factors. Similar gene family members were expressed in both the FAZ and the LAZ following flower or leaf removal, respectively, suggesting a similar regulation of the abscission process of these organs with few exceptions. This provides a significant improvement in our understanding of the abscission process.

We have utilized the RNA-Seq data for the development of an AZ-specific microarray chip. The AZ microarray is more comprehensive than other commercially available arrays, having more transcripts with multiple probes and probes designed in antisense direction, which might be further used to explore the roles of NATs. The AZ-specific microarray can be used for further examination of detailed gene expression in various abscission stages, and is applicable to other transcriptomic studies in tomato. The AZ-specific microarray chip provides a cost-effective approach for analysis of multiple samples in a rapid succession. Hence, this study can serve as a foundation for characterization of candidate genes, which would not only provide novel insights into understanding of the AZ development, early and late abscission events, but also will provide resources for improved tomato breeding for preventing abscission and for specific manipulation of flower or leaf abscission for horticultural uses.

## Sequence deposition

NGS data is submitted to the NCBI sequence read archive (NCBI-SRA) under the study ID: PRJNA192557. Individual SRS ID = SRS399193 entitled with FAZ transcriptome analysis of VF-36, and SRS401162 entitled with LAZ transcriptome analysis of VF-36. The AZ-specific microarray (AMADID: 043310) was validated and used to analyze the FAZ, flower NAZ and LAZ at various time points and deposited in the NCBI Gene Expression Omnibus (GEO) databases (http://www.ncbi.nlm.nih.gov/geo/) under the following locators: GSE45355; GSE45356; GSE64221. These data will be released for public access upon acceptance of this publication.

## Author contributions

SS, SM and SP were responsible for the conception, design of the experiments and interpretation of data for work. SS, RM and NK were responsible for acquisition of the data and the bioinformatics analysis. SS, BK and ShS performed the laboratory experiments and analyses. SS, RJ, and MT were responsible for the design of the microarray chip. SS, SP, JR and SM were responsible for final approval of the version to be published.

## Funding

This work was supported by the United States-Israel Binational Agricultural Research and Development Fund (BARD) [grant number US-4571-12C to SM, MT and SP], and the Chief Scientist of the Israeli Ministry of Agriculture Fund [grant number 203-0898-10 to SM and SP].

### Conflict of interest statement

The authors declare that the research was conducted in the absence of any commercial or financial relationships that could be construed as a potential conflict of interest. The reviewer Autar Krishen Mattoo declares that, despite being affiliated with the same institute as the author Mark L. Tucker, the review process was conducted objectively.

## Abbreviations

AREs: auxin-responsive elements
ARF: auxin response factor
Aux/IAA: auxin resistant/indole-3-acetic acid-inducible
AZ: abscission zone
bHLH: basic helix–loop–helix
Bl: blind
BLAST: basic local alignment search tool
bZIP: basic leucine zipper
Cel: cellulase
CTR: constitutive triple response
DEG: differentially expressed genes
EIL: ethylene insensitive-like
EIN: ethylene insensitive
ERF: ethylene responsive factor
EXP: expansin
FAZ: flower abscission zone
GH: Gretchen Hagen3
GO: gene ontology
IAA: indole-3-acetic acid
JA: jasmonic acid
KEGG: Kyoto encyclopedia of genes and genomes
LAX: auxin influx carrier (Like Aux)
LAZ: leaf abscission zone
LBD1: lateral organ boundaries domain protein1
Ls: lateral suppressor
MAPKK: mitogen- activated protein kinase kinase
MAPKKK: MAPKK Kinase
1-MCP: 1-methylcyclopropene
NAT: naturally occurring antisense transcripts
NAZ: non-AZ
NCBI: national center for biotechnology information
NGS: next generation sequencing
OFP: ovate family protein
PG: polygalacturonase
PIN: pin-formed
PE: paired end
QC: quality check
qRT-PCR: quantitative real time PCR
RNA-Seq: RNA sequencing
SAM: shoot apical meristem
SAUR: small auxin up-regulated RNA
TAGL: tomato AGAMOUS-like
TAPG: tomato abscission polygalacturonase
TF: transcription factor
WUS: WUSCHEL-related homeobox-containing protein
XTH: xyloglucan endotransglucosylase-hydrolase
ZF: zinc finger.
