# Supplementary material for: De novo Transcriptome Sequencing and Development of Abscission Zone-Specific Microarray as a New Molecular Tool for Analysis of Tomato Organ Abscission
Source: Front Plant Sci. 2016 Jan 14;6:1258. doi: 10.3389/fpls.2015.01258 (PMC4712312; doi:10.3389/fpls.2015.01258)
Supplement: Supplementary file 1 [file Table1.DOCX]

**Table S1:** List of primers and genes selected for the qRT-PCR analysis. F = forward; R = reverse.

| **Serial No** | **Gene Name** | **Gene ID** | **Primer sequence (5’ to 3’)** | **Tm (°C)** | **Size (bp)** |
| --- | --- | --- | --- | --- | --- |
| 1 | *Actin*_F | Solyc03g078400 | GTGTTGGACTCTGGTGATGG | 60 | 216 |
| 2 | *Actin*_R |  | GTAGTCAAGAGCCACATAAGC | 60 |  |
| 3 | *LeACO*_F | Solyc06g060070 | TGGCTCAAATTTCCAAAG | 53.2 | 197 |
| 4 | *LeACO*_R |  | TTGAACACTTCTTCTCTTTCCAA | 55.3 |  |
| 5 | *SlMYB21*_F | Solyc02g067760 | AGGAAAGGGCCTTGGACTAT | 57.3 | 158 |
| 6 | *SlMYB21*_R |  | CGAAGATAATTTAGCCATCGG | 55.9 |  |
| 7 | *SlLAX4* _F | Solyc10g076790 | GAGCTGGACTGCATACCTCA | 59.4 | 178 |
| 8 | *SlLAX4* _R |  | GCTTTCCAGTAAGGACCCAA | 57.3 |  |
| 9 | *Sl-IAA24*_F | Solyc09g083290 | TTCTGAGACTGTTGATTTGAAGC | 60 | 167 |
| 10 | *Sl-IAA24*_R |  | GTGGCCAACCCACAACTT | 60 |  |
| 11 | *NPR1-like protein_*F | Solyc10g079750 | CGATTGCACAACAAATAGGG | 60 | 122 |
| 12 | *NPR1-like protein*_R |  | GGTCAAATTCACCATGCCTA | 60 |  |
| 13 | *Sl-ARF18*_F | Solyc01g096070 | TTCTGCAAGATGGTGAGGAG | 60 | 103 |
| 14 | *Sl-ARF18*_R |  | TCCCTTCACCTTCCAATGAT | 60 |  |
| 15 | *GH3-15*_F | Solyc12g005310 | ATGCAGTGAGCCACTTGATG | 60 | 121 |
| 16 | *GH3-15*_R |  | GTTGAGCCATTCACGTTGAC | 60 |  |
| 17 | *LOB domain protein*_F | Solyc02g086480 | CAAGTTATACGGCTTCAGAAAGAA | 60 | 145 |
| 18 | *LOB domain protein*_R |  | CAGGCTGAGGGTTAGGGTTA | 60 |  |
| 19 | *Peroxidase1*_F | Solyc10g076210 | TGCATTCTGATCAAGCACTGT | 60 | 147 |
| 20 | *Peroxidase1*_R |  | GGCCTTCATTTCCAGTGAGT | 60 |  |
