# Supplementary material for: De novo Transcriptome Sequencing and Development of Abscission Zone-Specific Microarray as a New Molecular Tool for Analysis of Tomato Organ Abscission
Source: Front Plant Sci. 2016 Jan 14;6:1258. doi: 10.3389/fpls.2015.01258 (PMC4712312; doi:10.3389/fpls.2015.01258)
Supplement: Supplementary file 12 [file Image1.PDF]

De novo transcriptome sequencing and development of abscission zone-specific microarray as a new molecular tool for analysis of tomato organ abscission

Srivignesh Sundaresan, Sonia Philosoph-Hadas, Joseph Riov, Raja Mugasimangalam, Nagesh A Kuravadi, Betina Kochanek, Shoshana Salim, Mark L. Tucker and Shimon Meir*

***Correspondence:** Corresponding Author: shimonm@volcani.agri.gov.il

Supplementary Figures

A

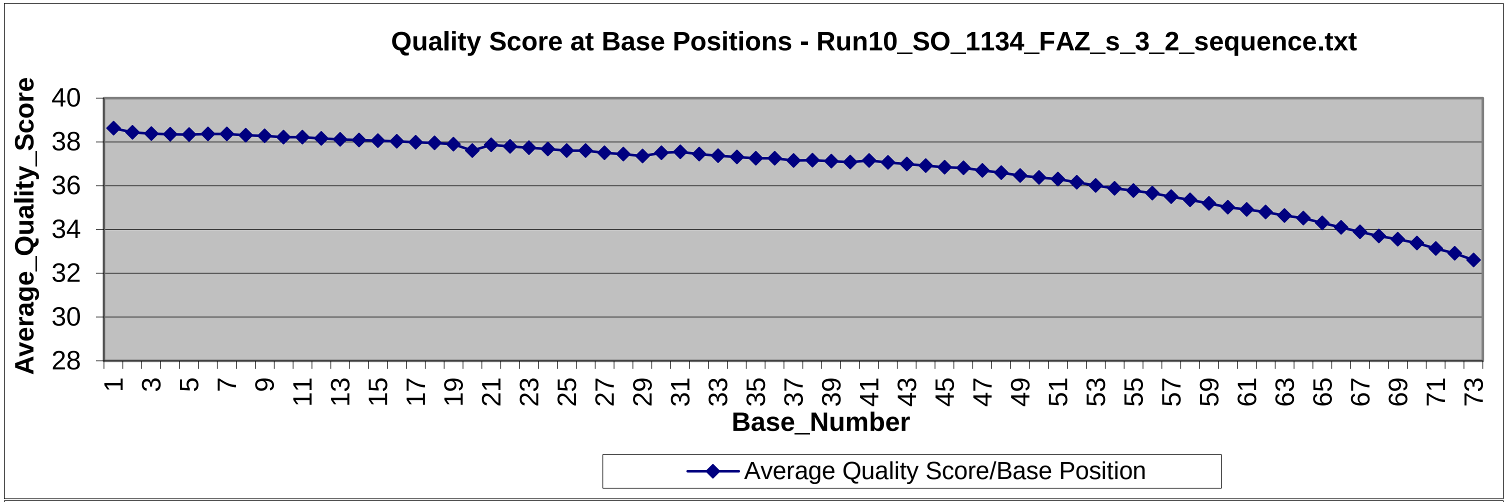

B

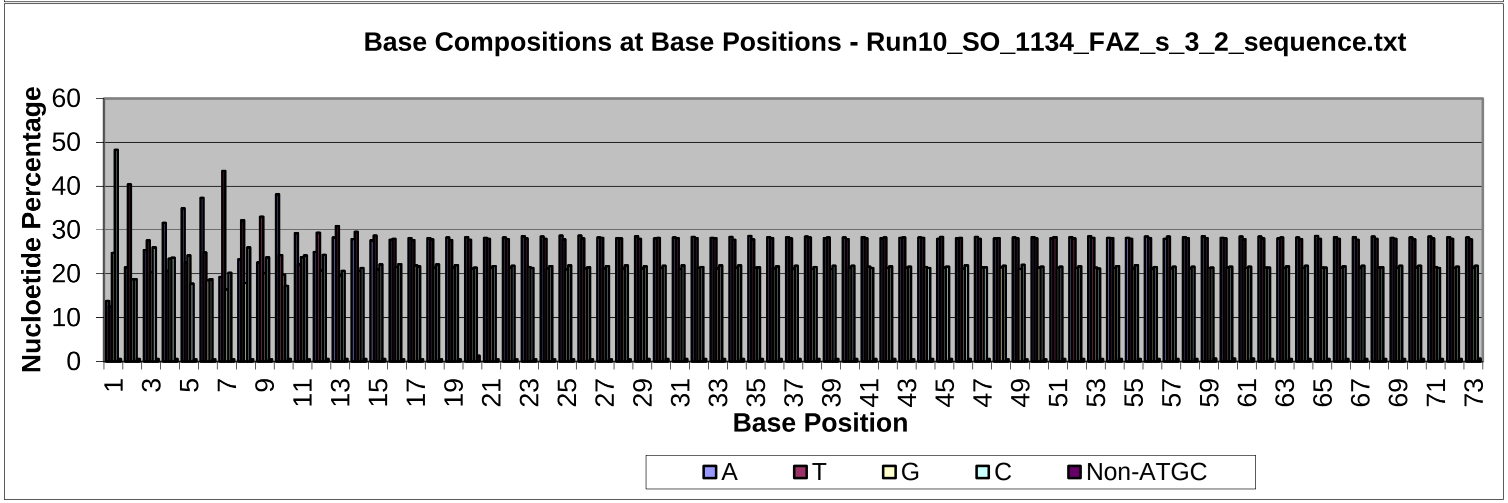

Figure S1. Quality score (A) and base composition (B) at base positions for the tomato FAZ pooled samples.

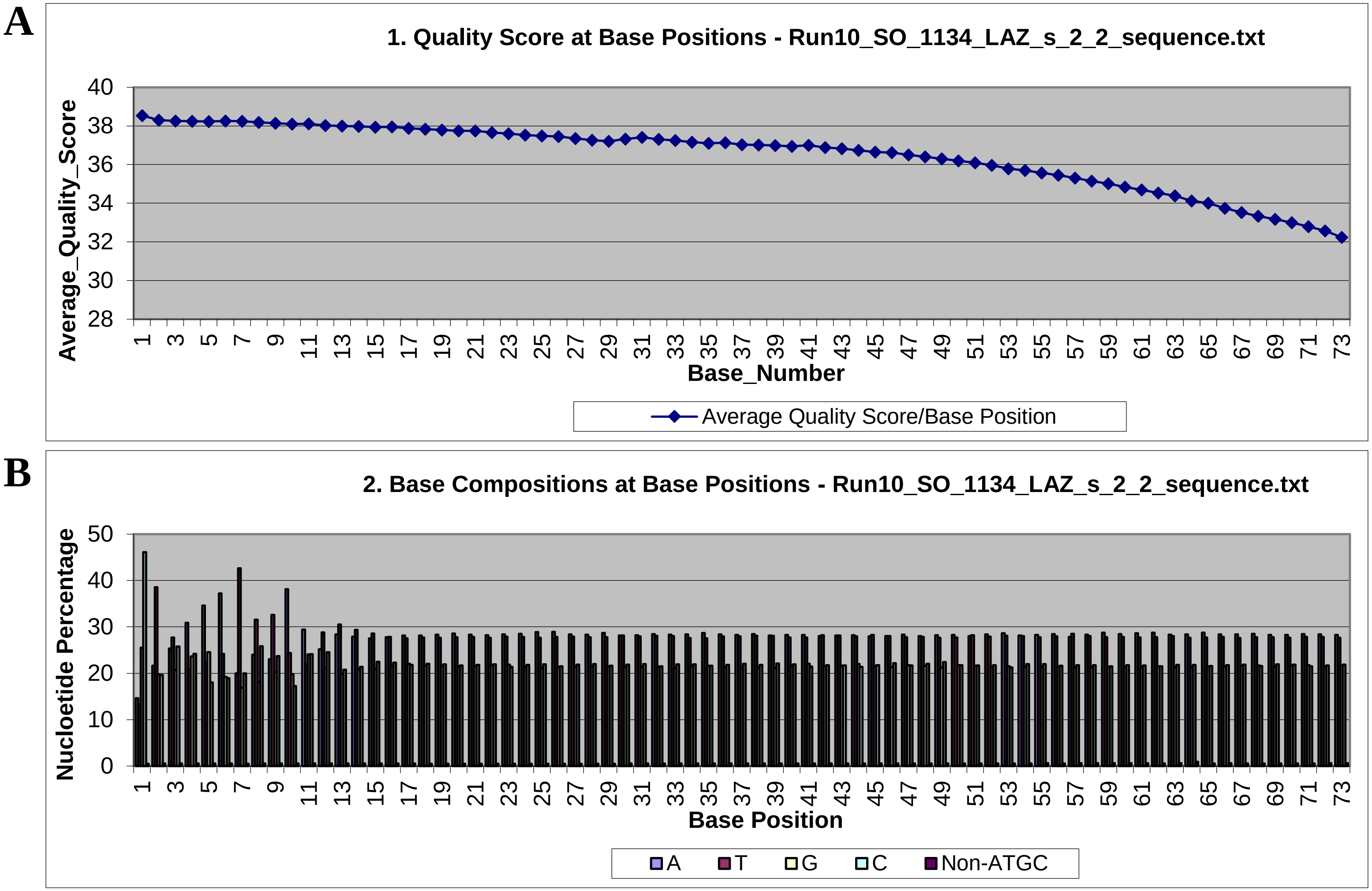

Figure S2. Quality score (A) and base composition (B) at base positions for the tomato LAZ pooled samples.

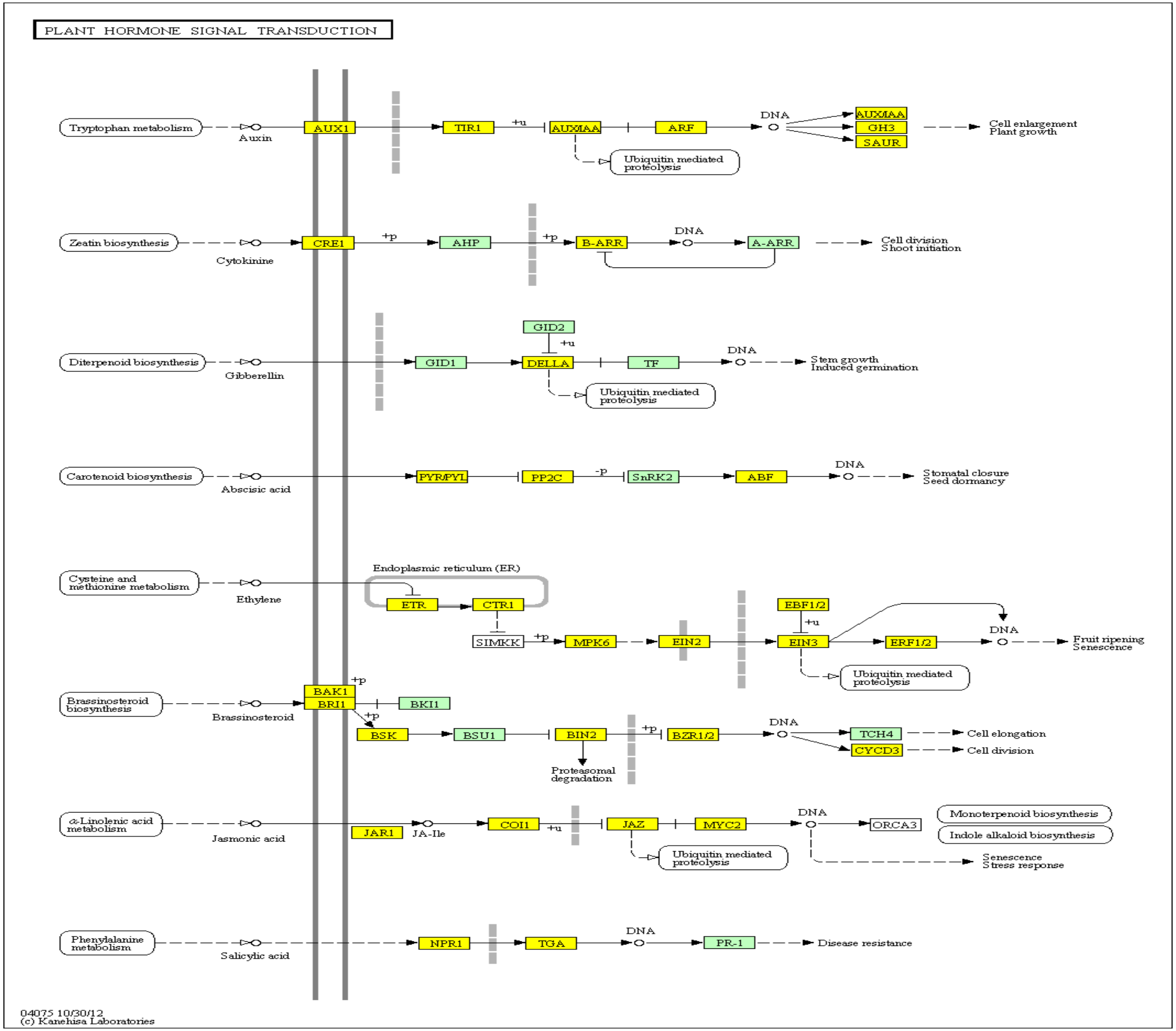

Figure S3. Kyoto Encyclopedia of Genes and Genomes (KEGG) of hormonal signal transduction pathways in the tomato FAZ samples. The boxes in yellow are the genes present and the boxes in green represent genes not covered in the transcriptome.

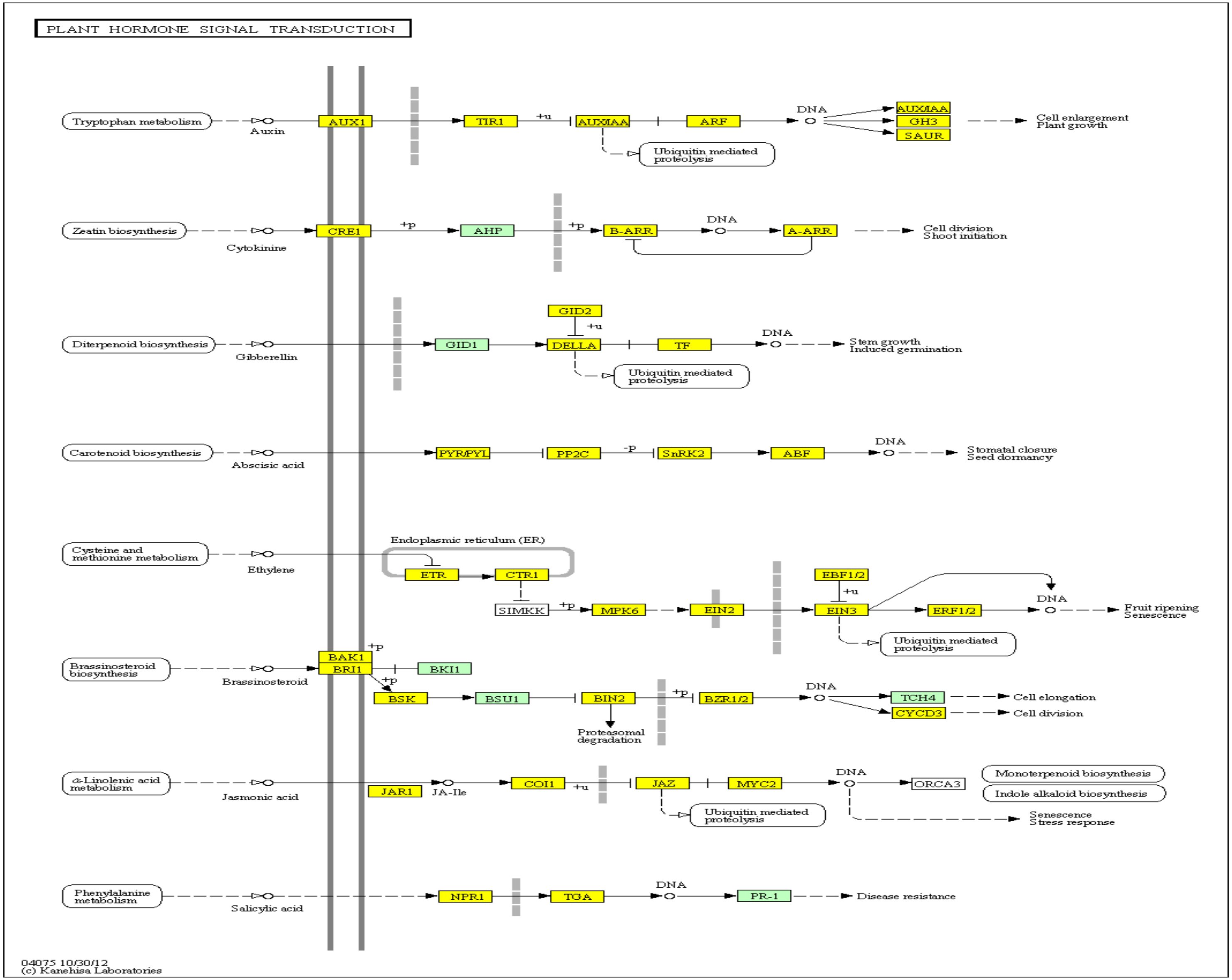

Figure S4. Kyoto Encyclopedia of Genes and Genomes (KEGG) of hormonal signal transduction pathways in the tomato LAZ samples. The boxes in yellow are the genes present and the boxes in green represent genes not covered in the transcriptome.

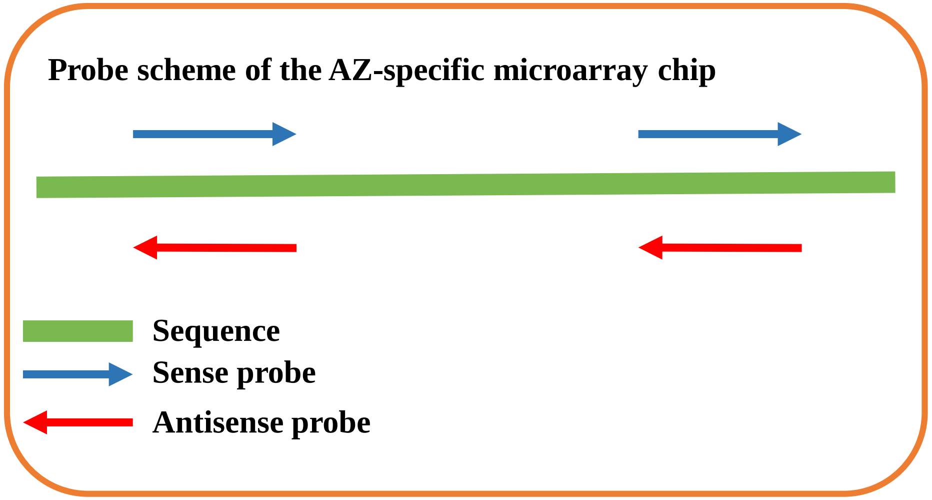

Figure S5. Schematic illustration of the probe design in the AZ-specific microarray chip. The arrows indicate the direction of the probes, sense (blue) or antisense (red).
